# Chloroplast–Thylakoid Organisation Is More Important than Carotenoid Accumulation for Optimum Photosynthetic Quantum Yield and Carbon Gain in Variegated *Epipremnum aureum*

**DOI:** 10.3390/cells15060514

**Published:** 2026-03-13

**Authors:** Renan Falcioni, Werner Camargos Antunes, Marcelo Luiz Chicati, José Alexandre M. Demattê, Marcos Rafael Nanni

**Affiliations:** 1 Graduate Program in Agronomy, State University of Maringá, Av. Colombo, 5790, Maringá 87020-900, PR, Brazil; wcantunes@uem.br (W.C.A.); mlchicati@uem.br (M.L.C.); mrnanni@uem.br (M.R.N.); 2 Department of Soil Science, Luiz de Queiroz College of Agriculture, University of São Paulo, Av. Pádua Dias, 11, Piracicaba 13418-260, SP, Brazil; jamdemat@usp.br

**Keywords:** chlorophyll a fluorescence, chloroplasts and thylakoids, electron microscopy, leaf optical properties, photochemical and carboxylative efficiencies

## Abstract

**Highlights:**

**What are the main findings?**

- CO_2_ yield per absorbed photon and carbon gain track chloroplast–thylakoid/grana organisation more than carotenoid accumulation.
- ‘Neon’ overloads fewer photosystem II centres (low quenching, carbon-poor); ‘Jade’ spreads excitation (carbon-rich, cool), while ‘Golden’ maximises water-use efficiency.

**What are the implications of the main findings?**

- Thylakoid abundance sets a structural ceiling for photoprotection and CO_2_ yield, so pigment ratios alone can be misleading.
- Hyperspectral, fluorescence and thermal imaging can non-destructively phenotype leaves, linking chloroplast structural state to photosynthetic efficiency, canopy temperature and water-use economy.

**Abstract:**

Coloured and variegated leaves are common in shade-tolerant ornamentals. However, it remains unclear whether their photosynthetic performance is determined mainly by pigment abundance or by the organisation of chloroplasts and thylakoids. We tested this in three *Epipremnum aureum* phenotypes (‘Neon’, ‘Golden’ and ‘Jade’) that share a genetic background but contrast in leaf colour, chloroplast density and thylakoid membrane abundance. Plants were grown in a greenhouse and assessed by hyperspectral and thermal imaging, infrared gas exchange analysis, chlorophyll a fluorescence measurements, and structural, ultrastructural and biochemical analyses. Traits were integrated by principal component analysis, with the quantum yield of CO_2_ assimilation per absorbed photon (αCO_2,abs_) as the response variable. ‘Neon’ leaves had high specific leaf area and approximately 55% lower maximum Rubisco carboxylation (Vc_MAX_) and electron transport capacity (J_MAX_) than ‘Jade’, as well as reduced chloroplast and thylakoid abundance and warmer canopies, despite carotenoid enrichment. JIP-test parameters and fluorescence light–response curves showed high absorption and dissipation per PSII reaction centre, elevated excitation pressure, modest non-photochemical quenching (NPQ), low αCO_2,abs_, small carbohydrate pools and low intrinsic water-use efficiency. ‘Jade’ leaves developed thick mesophyll with dense chloroplast populations, extensive thylakoid networks, highest NPQ, cool canopies and large carbohydrate reserves, whereas ‘Golden’ leaves combined thin laminae and intermediate chloroplast–thylakoid organisation with early light saturation of CO_2_ assimilation and the highest intrinsic water-use efficiency. Principal component analysis revealed a structural axis of chloroplast and thylakoid organisation that better predicted αCO_2,abs_, net carbon gain and canopy temperature than pigment abundance. In variegated *E. aureum*, ‘photon economy’ is therefore governed primarily by chloroplast and thylakoid membrane organisation and abundance rather than by carotenoid accumulation.

## 1. Introduction

Leaves are the primary organs for light capture and carbon assimilation, yet in many woody evergreens and shade-tolerant vines they also display coloured or variegated laminae. Variegated leaves arise when chloroplast biogenesis is spatially disrupted, producing sectors with contrasting pigment contents and optical properties [1,2,3,4]. Recent genetic and multi-omics studies across cereals, woody ornamentals and fruit trees have shown that such mosaics often reflect local defects in chlorophyll biosynthetic enzymes, thylakoid assembly or plastid–nuclear signalling, leading to stable patches of chlorophyll-deficient tissue embedded within photosynthetically competent green sectors [5,6,7,8]. In *Epipremnum aureum*, ‘Golden Pothos’ impaired expression of EaZIP, which encodes a Mg-protoporphyrin IX monomethyl ester cyclase and generates long-living albino and variegated lines [9]. Likewise, *Arabidopsis GENOMES UNCOUPLED* (*GUN*) mutants, such as *gun1* and *gun4*, disrupt plastid-to-nucleus signalling and Mg-chelatase-dependent tetrapyrrole biosynthesis, respectively, and develop chlorophyll-poor chloroplasts with impaired thylakoid development, which are phenotypes that resemble the stable pale and variegated sectors observed in many ornamental plants [2,3,10]. These observations fit within a broader framework in which chloroplast biogenesis is controlled by coordinated nuclear–plastid gene expression and transcriptional regulators, including *GOLDEN’2-LIKE* (*GLK*) transcription factors and ROS-dependent retrograde pathways [4,8,11].

The functional meaning of leaf variegation remains debated. In many mutants and chimaeras, pale sectors harbour abnormal or photosynthetically compromised chloroplasts, and variegated seedlings and mature plants typically show reduced whole-leaf photosynthetic rates and growth relative to fully green conspecifics [5,12,13]. More generally, mechanistic work on fluctuating-light responses and non-photochemical quenching indicates that dynamic adjustments in pigment–protein complexes and thylakoid organisation can attenuate excitation pressure and limit chronic photodamage under stressful light regimes [14,15]. In parallel, elevational clines in leaf variegation suggest that its frequency and intensity covary with local climate and soil moisture availability [12]. Pale or white sectors often accumulate carotenoids, flavonoids or ROS-scavenging enzymes, and mechanistic work now links the GLK-dependent programmes of chloroplast biogenesis directly to carotenoid biosynthesis and antioxidant capacity [4,11,13]. However, most studies have focused either on the molecular mechanisms underlying chloroplast deficiency or on leaf-level photosynthesis and growth, rather than integrating pigment distributions, chloroplast ultrastructure, photochemistry and carbon allocation within a single framework [5,6,8]. Moreover, organelle-centred perspectives increasingly emphasise that plastids operate in concert with mitochondria and peroxisomes, such that disruptions in chloroplast and thylakoid biogenesis are expected to propagate to respiratory metabolism and peroxisome-linked redox and antioxidant networks, with downstream consequences for water-use efficiency and leaf thermal balance [16,17,18].

Variegated and otherwise colourful leaves have been exploited as model systems for optical phenotyping. UV–VIS–NIR–SWIR hyperspectral and chlorophyll a fluorescence (ChlF) sensors can resolve steep pigment gradients, detect PSII injury and predict biochemical constituents in leaves, and recent work emphasises the integrative character of hyperspectral reflectance as a proxy for metabolic state [13,19,20,21]. Yet, these approaches rarely quantify how variation in chloroplast number, thylakoid/grana architecture, stomatal traits and carbohydrate status jointly determines hyperspectral signatures and chlorophyll fluorescence (ChlF) responses (e.g., OJIP transients and fluorescence light–response curves) [13,22,23], or how these traits co-vary across stable gradients of variegation. As a consequence, we still lack a mechanistic description of how structural adjustments in variegated leaves rebalance photon capture, energy dissipation, water use and carbon storage. In this context, we quantify photon economy as the quantum yield of CO_2_ assimilation per absorbed photon (αCO_2,abs_; mol CO_2_ mol^−1^ photons absorbed) and relate this metric directly to leaf absorptance and multi-scale structural and photochemical traits [4,24,25].

*Epipremnum aureum* (Linden & André) G.S. Bunting plant is ideally suited to address this gap. It is a clonal, shade-tolerant hemiepiphyte widely cultivated indoors, where leaves often operate close to the light compensation point under low or diffuse irradiance [26]. In addition to naturally variegated ‘Pothos’, tissue culture has yielded stable lime-coloured and fully green lines that differ strongly in apparent chlorophyll content yet can be propagated under identical conditions, providing a genetically constrained gradient in chloroplast abundance and pigment patterning [25].

Here, using three *E. aureum* cultivars (‘Neon’, ‘Golden’ and ‘Jade’), we quantify how photochemical energy conversion translates into carboxylative CO_2_ assimilation along a genetically constrained gradient in chloroplast abundance and thylakoid organisation [5,27]. We combined hyperspectral reflectance with gas exchange and chlorophyll fluorescence diagnostics, together with quantitative anatomy, transmission electron microscopy, stomatal traits, pigment and phenolic profiles, cell-wall chemistry and carbohydrate pools. Principal component and correlation analyses were then used to connect traits from chloroplast ultrastructure to whole-leaf function, in line with recent multi-trait and spectral-ecophysiological approaches that seek latent axes linking structure, optics and metabolism [19,20,28]. We tested three related hypotheses: that variation in leaf colour and variegation reshapes the within-leaf light distribution (H_1_) [12,21,29], that chlorophyll-poor leaves compensate via photochemical adjustment and carbon allocation to carotenoids and phenolics (H_2_) [6,11,30], and that these adjustments provide feedback of stomatal behaviour, water-use efficiency and carbohydrate storage near the light compensation point (H_3_) [24,25]. Our unique contribution is the integration of hyperspectral optics, gas exchange, fluorescence kinetics and quantitative ultrastructure within a single *E. aureum* system to test whether chloroplast–thylakoid organisation, rather than carotenoid enrichment alone, predicts αCO_2,abs_, carbon gain and canopy temperature. This moves beyond purely correlative hyperspectral phenotyping by explicitly linking spectral signatures to chloroplast architecture and to biochemical capacity derived from A–C_i_ modelling.

## 2. Material and Methods

### 2.1. Plant Material and Experimental Design

*Epipremnum aureum* (Linden & André) G.S. Bunting plants with contrasting leaf colours were grown in a greenhouse at the Botanic Garden, State University of Maringá (Maringá, PR, Brazil). We used three commercial ornamental cultivars widely marketed for indoor environments: ‘Neon’ (uniform lime-green leaves with low apparent chlorophyll), ‘Golden’ (variegated leaves with green, yellow and albino sectors) and ‘Jade’ (uniformly dark-green leaves). Rooted cuttings of each cultivar were grown under identical greenhouse conditions and transplanted into 2 L plastic pots filled with a peat-based commercial substrate (MecPlant^®^, Telêmaco Borba, PR, Brazil) and grown to marketable size under standard commercial ornamental production practices for subtropical southern Brazil.

The greenhouse was covered with clear plastic film and side curtains and received only natural daylight (no supplementary lighting or blackout). During the growth period, the air temperature reflected ambient subtropical conditions typical of Maringá city (approximately 24–30 °C during the day and 18–22 °C at night), which fall within the recommended range for *E*. *aureum* cultivation. The relative humidity was maintained between 60% and 80%. The plants were irrigated once daily for drainage with Hoagland nutrient solution (pH 5.5), and the pots were allowed to drain freely.

Leaf canopy development and the expression of contrasting leaf colours occurred under the natural day length and light regime at this latitude, which is suitable for this shade-tolerant hemiepiphytic species commonly, which is typically used as an indoor ornamental. For each cultivar, fully expanded, non-senescent leaves from the mid-canopy were sampled from ten independent plants (biological replicates, *n* = 10 per cultivar) and allocated for biochemical, optical, physiological and anatomical measurements as described below. The experiment followed a completely randomised design with leaf-colour phenotype (‘Neon’, ‘Golden’, ‘Jade’) as the fixed factor.

### 2.2. Hyperspectral and Thermal Optical Leaf Properties

Leaf optical properties were characterised in situ on the attached laminae of the same plants used for physiological measurements. Directional–hemispherical reflectance and transmittance spectra (350–2500 nm) were acquired with a FieldSpec^®^ 3 spectroradiometer (Analytical Spectral Devices, ASD Inc., Longmont, CO, USA) coupled to an ASD Contact PlantProbe^®^ (Analytical Spectral Devices, ASD Inc., Longmont, CO, USA) leaf clip. The instrument combines a 512-element silicon photodiode array for the visible–near-infrared region (350–1000 nm) and two thermoelectrically cooled graded-InGaAs photodiodes for the short-wave infrared (SWIR; 1000–1800 and 1800–2500 nm; i.e., the shorter-wavelength portion of the infrared region) [31].

For each measurement, the internal quartz–tungsten–halogen source of the PlantProbe^®^ illuminated either the adaxial or the abaxial surface. Reflectance was obtained by normalising the leaf radiance to that of a Spectralon^®^ white reference panel (Labsphere Inc., North Sutton, NH, USA). Transmittance was recorded with the detector positioned behind the lamina in the same optical geometry and normalised to the radiance transmitted through the empty clip. Spectra were collected at 1 nm sampling intervals and averaged to obtain one reflectance (R_λ_) and one transmittance (T_λ_) spectrum per leaf surface. Spectral absorptance (A_λ_; unitless fraction of incident radiation absorbed) was calculated as A_λ_ = 1 − R_λ_ − T_λ_ after correction for the dark current [31]. Here, we use the radiometric term ‘absorptance’ for the fraction absorbed (1 − R − T) and reserve ‘absorbance’ for spectrophotometric optical density measurements. These R, T and A spectra were subsequently used as inputs for multivariate analyses of spectral variation among the cultivars.

Long-wave infrared thermal images were obtained with a FLIR Vue Pro radiometric camera (FLIR Systems, Danderyd, Sweden; spectral band 7.5–13.5 µm) mounted at a fixed distance and perpendicular to the plant canopy. Images were captured for the same plants used in the gas-exchange measurements. The leaf emissivity was set to 0.98, and images were acquired at a fixed distance under stable greenhouse conditions. Thermal images were used as a non-contact indicator of canopy surface temperature (an emergent property of leaf energy balance) and were interpreted together with gas-exchange variables rather than as a possible correlation of PSII or leaves’ heat dissipation.

### 2.3. Biochemical Analyses

From each plant, leaf discs (≈1 cm^2^) were collected from fully expanded mid-canopy laminae and processed immediately. Photosynthetic and non-photosynthetic pigments were extracted following the chloroform–methanol partitioning protocol of Gitelson & Solovchenko (2018) [32], with minor adaptations. The discs were ground in 2 mL microtubes with chloroform:methanol (2:1, *v*/*v*) containing a small excess of CaCO_3_. After complete extraction, distilled water corresponding to 20% of the final volume was added to promote phase separation, and the tubes were centrifuged at 15,000 rpm (14,000× *g*; model K14-1215, Kasvi, Curitiba, PR, Brazil) for 5 min.

The lower chloroform-rich phase, containing total chlorophylls and carotenoids, and the upper methanol–water phase, containing non-chloroplastidic pigments and phenolic compounds, were transferred to UV-transparent 96-well microplates (200 µL well^−1^) and read in a UV–VIS microplate reader (Biochrom Asys UVM-340; Biochrom Ltd., Cambridge, UK). Absorbance of the chlorophyll–carotenoid phase was recorded at 470, 652 and 665 nm, and chlorophyll a, chlorophyll b, total chlorophyll (Chla+b) and total carotenoids were calculated using the equations of Gitelson and Solovchenko (2018) [32]. Data were expressed on both an area basis (mg m^−2^, using the sampled leaf area) and a dry-mass basis (mg g^−1^), with the reader software correcting for optical path length.

Flavonoids in the methanol–water phase were quantified at 358 nm, and anthocyanins were quantified after slight acidification of this phase (final 0.1% HCl) at 530 nm, using the specific absorption coefficients reported by Gitelson & Solovchenko (2018) [32] and cyanidin-3-glucoside as the reference standard. Total soluble phenolic content was determined in aliquots of the methanolic phase using the Folin–Ciocalteu reagent and expressed as mg gallic acid equivalents (GAE) g^−1^ dry mass. Antioxidant capacity was estimated by the DPPH radical-scavenging assay in the same microplate reader and expressed as the percentage of DPPH reduced relative to reagent blanks.

### 2.4. Cell Wall Composition Analyses

Protein-free cell wall (PFCW) material was prepared from dried, ground *E*. *aureum* leaf tissue following Moreira-Vilar et al. (2014) [33], with adaptations described by Falcioni et al. (2025) [34]. Approximately 150 mg of dry powder per sample was placed in 2 mL microtubes and subjected to sequential washes to remove soluble metabolites and proteins: five washes with 50 mM potassium phosphate buffer (pH 7.0), five with the same buffer containing Triton X-100, four with 1 M NaCl, four with distilled water and three with acetone. After each wash, the samples were centrifuged at ≈15,000 rpm (14,000× *g*; model K14-1215, Kasvi, Curitiba, PR, Brazil) for 3 min. The final pellets were dried at 60 °C for 24 h and considered PFCW.

#### 2.4.1. Lignin Quantification

Lignin in the PFCW was quantified by the acetyl bromide soluble lignin (ABSL) assay according to Moreira-Vilar et al. (2014) [33], adapted to microplates as in Falcioni et al. (2025) [34]. For each sample, 10 mg of PFCW was incubated with 130 µL of freshly prepared acetyl bromide solution (25% *v*/*v* in glacial acetic acid) at 70 °C for 30 min. After cooling on ice, 0.24 mL of 2 M NaOH, 0.02 mL of 5 M hydroxylamine-HCl and 1.6 mL of glacial acetic acid were added to complete lignin solubilisation. Tubes were centrifuged (≈1400× *g*, 5 min), and 200 µL of the supernatant was dispensed into UV-transparent 96-well plates. Absorbance at 280 nm was read in the FlexStation 3 microplate reader (Molecular Devices LLC., San Jose, CA, USA), and lignin concentration was calculated from an alkali lignin calibration curve using an extinction coefficient ε = 22.1 L g^−1^ cm^−1^. The results were expressed as mg lignin g^−1^ PFCW and were later converted to a dry-mass basis for comparison with other biochemical traits.

#### 2.4.2. Cellulose Quantification

Cellulose content was determined in the same PFCW fractions using the anthrone colourimetric assay adapted for microplates [34]. Aliquots of 10 mg PFCW were incubated with an acetic acid:nitric acid (70:30) mixture at 70 °C for approximately 1 h to remove non-cellulosic polysaccharides, washed repeatedly with distilled water and resuspended in concentrated H_2_SO_4_. Portions of this suspension were reacted with freshly prepared anthrone reagent in H_2_SO_4_, incubated in a boiling water bath, and cooled on ice, after which 200 µL of each reaction was transferred to 96-well plates. Absorbance at 620 nm was recorded using (Biochrom Asys UVM-340; Biochrom Ltd., Cambridge, UK), and the cellulose content was calculated from a glucose standard curve and expressed as glucose equivalents (mg glucose g^−1^ PFCW).

### 2.5. Soluble Sugar and Starch Extraction and Quantification

Soluble sugars and starch were extracted from lyophilised *E*. *aureum* leaf tissue [35,36], with minor modifications. Approximately 10 mg of dried powder from each sample was incubated with 1000 µL of pure methanol at 70 °C for 1 h. After incubation, the samples were centrifuged at 14,000× *g* for 10 min. From the supernatant, 700 µL was transferred to a new tube, 400 µL of deionised water and 500 µL of chloroform (CHCl_3_) were added, and the mixture was vortexed and centrifuged again to separate phases. A 1000 µL aliquot of the polar phase was collected and evaporated to dryness in a speed-vacuum concentrator and subsequently resuspended in assay buffer for soluble sugar determination.

The pellet remaining after the initial methanol extraction, enriched in insoluble carbohydrates, was washed three times with 80% (*v*/*v*) ethanol and used for starch determination. Starch was solubilised in hot KOH and hydrolysed enzymatically following Trethewey et al. (1998) [35]. Concentrations of glucose, fructose, sucrose and starch were determined by coupled enzymatic assays leading to NADH formation, and the absorbance was recorded at 340 nm in a microplate reader. Quantification of each metabolite was based on calibration curves constructed with known concentrations of the corresponding pure standards on the same 96-well plate. Results were expressed on a dry-mass basis (µmol g^−1^ or mg g^−1^ dry mass) for subsequent statistical analyses.

### 2.6. Gas-Exchange and Chlorophyll Fluorescence Measurements

#### 2.6.1. Light–Response (A–PAR) Curves

Gas exchange was measured on healthy, fully expanded mid-canopy leaves of ‘Neon’, ‘Golden’ and ‘Jade’ plants. An infrared gas analyser (LI-6800, LI-COR Biosciences, Lincoln, NE, USA) coupled with a Multiphase Flash™ fluorometer (LI-6800-01, LI-COR Biosciences, Lincoln, NE, USA) was used to measure net CO_2_ assimilation (A; µmol CO_2_ m^−2^ s^−1^), intercellular CO_2_ concentration (C_i_; mmol mol^−1^), stomatal conductance to water vapour (g_s_; mmol H_2_O m^−2^ s^−1^) and transpiration (E; mmol H_2_O m^−2^ s^−1^). Photosynthetic light—response (A–PAR) curves were obtained by stepping the PPFD from saturating irradiance down to darkness (2500, 2000, 1800, 1500, 1200, 1000, 800, 600, 400, 300, 200, 150, 100, 75, 50, 25, and 0 µmol photons m^−2^ s^−1^ in 17 steps; with each step lasting 50–70 s). Measurements were made under a red:blue light ratio of 90:10, a reference CO_2_ concentration of 400 µmol mol^−1^ in the sample chamber, a relative humidity close to 60%, a flow rate of 700 µmol s^−1^, a fan speed of 10,000 rpm and a leaf temperature of 25 °C. At each PPFD step, gas exchange was allowed to reach a steady state before recording A, C_i_, g_s_ and E, and fluorescence was measured simultaneously, as described in the chlorophyll a fluorescence section.

For each leaf, A–PAR curves were fitted with a non-rectangular hyperbola using a custom Python software version 3.10 (Python Software Foundation, Wilmington, DE, USA) and Excel (Microsoft Inc., Silicon Valley, CA, USA) routine (SciPy, least-squares). From the fits we obtained dark respiration in darkness (Rd), apparent quantum yield (α), maximum net CO_2_ assimilation at saturating irradiance (PN_MAX_), light compensation point (LCP) and light saturation point (LSP). Intrinsic water-use efficiency (iWUE) was calculated at 1200 µmol photons m^−2^ s^−1^ as A/g_s_. Electron transport rate (J) was used to estimate the ATP and NADPH supply under standard linear electron flow stoichiometry (J/4 for NADPH and 3J/8 for ATP); the corresponding values per unit CO_2_ fixed were also calculated. For Table 1 (see below), ATP and NADPH in the A–PAR block were derived from the maximum observed J along the light–response curve, whereas ETR in the fluorescence block refers to the operating value at 1200 µmol photons m^−2^ s^−1^.

#### 2.6.2. CO_2_-Response (A–C_i_) Curves and Photosynthetic Capacity

Photosynthetic A–C_i_ response curves were constructed on the same leaves used for A–PAR, using the LI-6800 in CO_2_ response mode with the Multiphase Flash™ fluorometer active. CO_2_–response curves (A–C_i_) were measured on the same leaves immediately after A–PAR curves whenever possible, or on equivalent leaves on the same plants. Leaves were exposed to saturating PPFD (1200 µmol photons m^−2^ s^−1^), and the reference CO_2_ concentration (C_a_) in the sample chamber was stepped from sub-ambient to supra-ambient values (400, 300, 200, 100, 50, 400, 600, 800, 1000, 1200, 1400, 1600, 1800, 2000 µmol mol^−1^ in 14 steps; 50 to 70 s each step). At each CO_2_ level, after the steady state was reached, the net CO_2_ assimilation (A; µmol CO_2_ m^−2^ s^−1^), intercellular CO_2_ concentration (C_i_; mmol mol^−1^), leaf temperature (Tleaf; °C), stomatal conductance to water vapour (g_sw_; mmol H_2_O m^−2^ s^−1^) and C_a_ (mmol mol^−1^) were recorded.

A–C_i_ curves were fitted with the three-limitation Farquhar–von Caemmerer–Berry model [37,38,39,40,41] using a custom Python software version 3.10 (Python Software Foundation, Wilmington, DE, USA) and Excel (Microsoft Inc., Silicon Valley, CA, USA) script based on the implementation of Sharkey et al. (2007) [41] and subsequent refinements. The script accounted for the temperature dependence of the Michaelis–Menten constants and of the CO_2_ compensation point and yielded, for each leaf, the maximum Rubisco carboxylation capacity (Vc_MAX_), the maximum electron-transport capacity supporting RuBP regeneration (J_MAX_), the triose-phosphate utilisation capacity (TPU) and day respiration (Rd*). From the fitted curves we also obtained the net CO_2_ compensation point (Γ), the CO_2_ saturation point (C_iSAT_) and the saturating assimilation rate (Asat), as reported in Table 1. From the fitted curves we also obtained Γ, C_iSAT_ and A_sat_, the latter retained for auxiliary calculations in Table 1 (see above).

Mesophyll conductance to CO_2_ (g_m_) and chloroplastic CO_2_ concentration (Cc) were inferred by numerically inverting the Farquhar model for each measured pair of A and C_i_. Stomatal conductance to CO_2_ (g_sc_) was calculated from stomatal conductance to water vapour (g_sw_/1.6), and an effective leaf-to-chloroplast conductance (g_Cc_) was derived from A, Ca and Cc. Mean values of Cc, g_m_, g_sw_ and g_Cc_ for each leaf were obtained by averaging over points with positive A and are summarised in Table 1. From the fitted A–C_i_ model, J_MAX_ was additionally converted into ATP- and NADPH-equivalent rates using the same simplified stoichiometric conversion adopted for the A–PAR analysis (NADPH equivalents = J_MAX_/4; ATP equivalents = 3 J_MAX_/8). These variables were used as model-derived descriptors associated with RuBP-regeneration capacity and were not interpreted as a strict mass-balance estimate of total ATP/NADPH demand allocated exclusively to carboxylation and photorespiration ranges.

FvCB parameters (Vc_MAX_, J_MAX_, TPU and Rd*) were estimated at the leaf level and used as inputs in the multivariate integration; canopy temperature was quantified empirically via thermal imaging rather than simulated.

#### 2.6.3. Fast OJIP Transients and JIP-Test Parameters

Fast chlorophyll a fluorescence induction curves (OJIP transients) were recorded on a separate set of leaves from the same plants using the LI-6800 (LI-COR Biosciences, Lincoln, NE, USA) in dark-adapted fluorescence mode. Leaves were dark-adapted for at least an overnight period in a humid chamber and then exposed to a saturating red pulse (≈15,000 µmol photons m^−2^ s^−1^, 625 nm, 1 s). Fluorescence was sampled from 20 µs to 1 s with a high-frequency acquisition scheme, yielding high-resolution OJIP curves.

The raw transients were exported and analysed with Biolyzer 4.0 (Laboratory of Bioenergetics, University of Geneva) to compute standard JIP-test parameters and energy-fluxes per reaction centre and per excited cross-section [42,43,44]. Difference kinetics for the ΔL, ΔK, ΔJ, ΔI and ΔH bands were calculated to characterise cultivar-dependent deviations in the OJIP transient, with emphasis on donor-side limitations and PSI acceptor-side behaviour. Energy pipeline models at the reaction-centre and cross-section scales were constructed from these parameters for each cultivar.

#### 2.6.4. Non-Steady-State Chlorophyll a Fluorescence Under Actinic Light

Chlorophyll a fluorescence parameters under actinic light were obtained simultaneously with gas exchange during the A–PAR protocol using the Multiphase Flash™ fluorometer (LI-6800-01, LI-COR Biosciences, Lincoln, NE, USA). Plants were dark-adapted overnight to determine the minimum fluorescence (F_0_) with a weak measuring beam and maximum fluorescence (Fm) with a saturating pulse; variable fluorescence (Fv = Fm − F_0_) and the maximum quantum efficiency of PSII photochemistry in the dark (Fv/Fm) were used to verify the absence of chronic photoinhibition.

During the A–PAR sequence, steady-state fluorescence (Fs), maximum fluorescence in the light (Fm′) and minimum fluorescence in the light (F_0_′) were recorded automatically by the LI-6800 at each irradiance step. From these signals, the maximum efficiency of PSII in the light (Fv′/Fm′), the effective quantum yield of PSII (Φ_PSII), the apparent electron transport rate (ETR), the quantum yield of CO_2_ assimilation (ΦCO_2_), non-photochemical quenching (NPQ), photochemical quenching (qP), the non-photochemical coefficient qN, the fraction of open PSII centres under the lake model (qL) and the complementary excitation-pressure index (1 − qL) were calculated following Maxwell & Johnson (2000) [45], Baker (2008) [46] and the LI-6800 manual. Values of these parameters at 1200 µmol photons m^−2^ s^−1^ were used for comparisons among the cultivars.

### 2.7. Microscopic Sample Preparation and Analyses

#### 2.7.1. Sample Preparation

Leaf samples (≈1 mm^2^) were collected from the median region of fully expanded ‘Neon’, ‘Golden’ and ‘Jade’ laminae. Pieces were immediately fixed in a modified Karnovsky solution [47] containing 2.5% (*v*/*v*) glutaraldehyde and 2% (*v*/*v*) paraformaldehyde in 0.05 M cacodylate buffer (pH 7.2) for 72 h at 4 °C. The same fixative was used for material destined for optical microscopy (OM), scanning electron microscopy (SEM) and transmission electron microscopy (TEM). After rinsing in cacodylate buffer, the samples were post-fixed for 6 h in 1% osmium tetroxide and 1.6% potassium ferrocyanide in 0.05 M cacodylate buffer, contrasted overnight in 0.5% aqueous uranyl acetate and dehydrated in an increased acetone series (30, 50, 70, 80, 90 and 100%, three changes in the final step).

A subset of dehydrated samples was reserved for SEM processing. The remaining material was infiltrated and embedded in Spurr’s low-viscosity epoxy resin and polymerised at 60 °C. Resin blocks were sectioned with an ultramicrotome (MTX Powertome X, Boeckeler Instruments RCM Products, Egham, UK) to obtain semithin (≈1.5 µm) and ultrathin (≈70 nm) sections using glass and diamond knives, respectively. All reagents were of electron-microscopy grade (Sigma, St. Louis, MO, USA; Electron Microscopy Sciences, Hatfield, PA, USA).

#### 2.7.2. Optical Microscopy Analyses

For OM, semithin sections were stained with 1% toluidine blue in borax buffer on a 70 °C hotplate for 30 s, rinsed in distilled water and mounted in synthetic resin. Sections were examined with a Leica ICC50 light microscope (Leica Microsystems, Wetzlar, Germany). Total leaf thickness and the thickness of the adaxial and abaxial epidermis and mesophyll were measured on digital micrographs using ImageJ software version 1.54p National Institutes of Health, Bethesda, MD, USA). Contrast enhancement and false-colour overlays used in the figures were generated in Image-Pro Plus^®^ version 7.1 (Media Cybernetics, Rockville, MD, USA).

To visualise native pigment distribution and mesophyll organisation, additional fresh transverse sections were obtained with a vibrating microtome (Leica VT1200 S, Leica Microsystems, Wetzlar, Germany). Sections were mounted in water and observed immediately under bright-field illumination.

#### 2.7.3. Stomatal and Guard-Cell Chloroplast Imaging

For stomatal observations, small pieces of abaxial epidermis were peeled from fresh leaves of each cultivar. For guard-cell viability staining and chloroplast imaging, peels were incubated for several minutes in an aqueous fluorescein diacetate and propidium iodide solution, rinsed briefly with buffer and mounted on slides. Bright-field and epifluorescence images were acquired sequentially with a EKB-2F epifluorescence microscope equipped with a FITC/GFP filter set (Eikonal Inc., São Paulo, SP, Brazil) so that viable guard cells fluoresced green, allowing the visualisation chloroplast-containing guard cells to be visualised. Stomatal pore morphology and guard-cell geometry were further examined on SEM preparations (see Section 2.7.4). Stomatal density, pore length, guard-cell width and the stomatal index on both leaf surfaces were quantified from OM and SEM images using Image-Pro Plus^®^ version 7.1 (Media Cybernetics, Rockville, MD, USA).

#### 2.7.4. Scanning Electron Microscopy Analyses

For SEM, dehydrated samples were subjected to critical-point drying in CO_2_ using a CPD-300 apparatus (Bal-Tec AG, Balzers, Liechtenstein). Dried samples were mounted on aluminium stubs, sputter-coated with gold (50 mA, 150 s) in an MED010 Bal-Tec evaporator (Bal-Tec AG, Balzers, Liechtenstein) and examined under an FEI Quanta 250 scanning electron microscope operated at 20 kV (FEI Company, Hillsboro, OR, USA). Cross-sections were used to visualise mesophyll architecture and intercellular-air-space distribution, whereas the leaf surfaces were imaged to assess the stomatal distribution and morphology. Quantitative measurements of tissue thickness, stomatal density and pore dimensions were obtained from calibrated digital images in Image-Pro Plus^®^ version 7.1 (Media Cybernetics, Rockville, MD, USA).

#### 2.7.5. Transmission Electron Microscopy Analyses

Ultrathin sections collected on 300-mesh copper grids were contrasted with 3% aqueous uranyl acetate for 40 min followed by lead citrate for 8 min, according to the methods of Reynolds (1963) [48]. Sections were examined under JEOL JEM-1400 transmission electron microscope (JEOL Ltd., Tokyo, Japan) operating at 80 kV and equipped with a digital camera. Mesophyll cells of the palisade parenchyma were analysed for chloroplast number, size and position, grana organisation, thylakoid stacking, plastoglobuli, starch grains, mitochondria and other organelles, aiming to identify these structures.

#### 2.7.6. Quantification of Anatomical and Ultrastructural Traits

Anatomical and ultrastructural traits were quantified for each cultivar from OM, SEM and TEM images using Image-Pro Plus^®^ and custom Python software version 3.10 (Python Software Foundation, Wilmington, DE, USA) and Excel (Microsoft Inc., Silicon Valley, CA, USA) routines. At the tissue level, adaxial and abaxial epidermal thickness, mesophyll thickness and total lamina thickness were measured on transverse sections. At the cellular level, the stomatal density and index, stomatal pore length and guard-cell width were determined separately for the adaxial and abaxial surfaces. At the chloroplast level, the chloroplast area per mesophyll cell profile, the number of chloroplasts per mesophyll cell profile, the number of thylakoid lamellae per granum, the grana thickness and the thylakoid density per unit chloroplast area were quantified from TEM micrographs. The frequency and size of plastoglobuli and starch granules were also recorded to aid qualitative interpretation of plastid differentiation.

### 2.8. Statistical Analyses

#### 2.8.1. Univariate Analyses

All variables were first checked for homogeneity of variances with Bartlett’s test [49]. Quantitative data were then analysed by one-way analysis of variance (ANOVA) with cultivar (‘Neon’, ‘Golden’, ‘Jade’) as the fixed factor. When the ANOVA was significant (*p* < 0.05 or 0.01), means were separated by Tukey’s post hoc test. Results are presented as means ± SE, and different letters or asterisks indicate significant differences among cultivars. When necessary, parameters obtained from curve fitting (A–PAR, A–C_i_ and fluorescence light–response curves) were analysed in the same way. Pearson’s correlation coefficients and cluster heatmaps were used to explore associations among selected traits. Statistical analyses were carried out in Statistica^®^ 10.0 (StatSoft Inc., Tulsa, OK, USA), SigmaPlot^®^ 10.0 (Systat Software Inc., San Jose, CA, USA), R version 2025.09.2 (R Core Team, R Foundation for Statistical Computing, Vienna, Austria) and custom Python software version 3.10 scripts (Python Software Foundation, Wilmington, DE, USA) were used for curve fitting, hyperspectral processing and data visualisation.

For traits measured on multiple sub-units per leaf (e.g., stomatal traits, chloroplast counts, and TEM-derived ultrastructural metrics), sub-samples were averaged to obtain a single value per leaf. Where more than one leaf per plant was measured for a given technique (e.g., hyperspectral optics), leaf values were averaged to the plant level prior to inferential statistics. Thus, the independent biological replicate for ANOVA/MANOVA/PCA was the plant (*n* = 10 per cultivar).

#### 2.8.2. Multivariate Analysis

To evaluate overall cultivar effects on sets of correlated traits, multivariate analysis of variance (MANOVA) was applied where appropriate, followed by principal component analysis (PCA) [50,51]. PCA was performed on centred and standardised data matrices using R and scikit-learn in Python Python software version 3.10 (Python Software Foundation, Wilmington, DE, USA). The analysis integrated variables grouped a priori into functional categories: (i) biochemical and molecular composition (pigments, phenolics, antioxidant capacity, soluble sugars, starch and cell-wall components); (ii) structure and ultrastructure (leaf and mesophyll thickness, chloroplast and thylakoid metrics); (iii) light–response parameters from A–PAR curves; (iv) carboxylative CO_2_-assimilation parameters from A–C_i_ curves; (v) chlorophyll a fluorescence parameters describing PSII energy partitioning under actinic light; (vi) JIP-test parameters from OJIP transients; and (vii) phenomenological PSII energy fluxes.

Principal components were retained based on eigenvalues and the proportion of total variance explained. PC1 and PC2 scores were used to describe the multivariate separation among cultivars and to examine how variation in the quantum yield of photosynthesis (αCO_2,abs_, defined as CO_2_ assimilation per absorbed photon flux) was related to the different functional categories. Mean PC1 and PC2 scores for each cultivar were compared by one-way ANOVA followed by Tukey’s test (*p* < 0.01). The relative contribution of each functional group to PC1 and PC2 was derived from the corresponding eigenvectors.

## 3. Results

### 3.1. Leaf Construction and Pigment Pattern Define Three Photon-Capture Strategies

**Figure 1:**
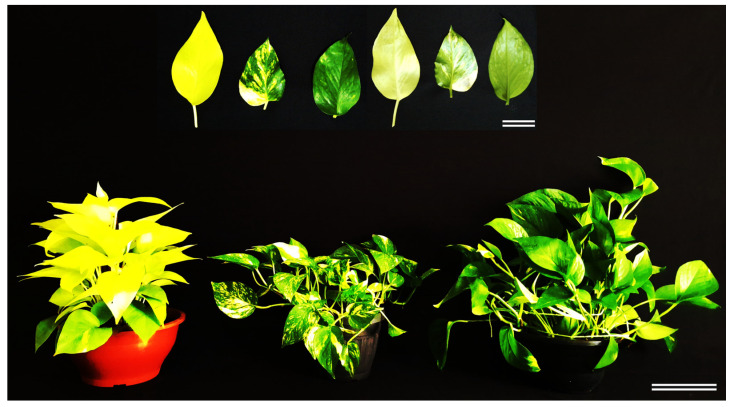
Whole-plant habit and leaf morphology of three leaf-colour phenotypes of *Epipremnum aureum* (Linden & André) G.S. Bunting. Representative ‘Neon’ (**left**), ‘Golden’ (**centre**) and ‘Jade’ (**right**) plants were photographed under standardised illumination against a black background. The inset shows representative fully expanded adaxial (**left**) and abaxial (**right**) leaves from each phenotype. Scale bars: main image (**bottom right**) = 15 cm; inset (**top right**) = 5 cm.

‘Neon’, ‘Golden’ and ‘Jade’ canopies are visually distinct, ranging from uniformly lime to strongly variegated and uniformly dark green (Figure 1).

**Figure 2:**
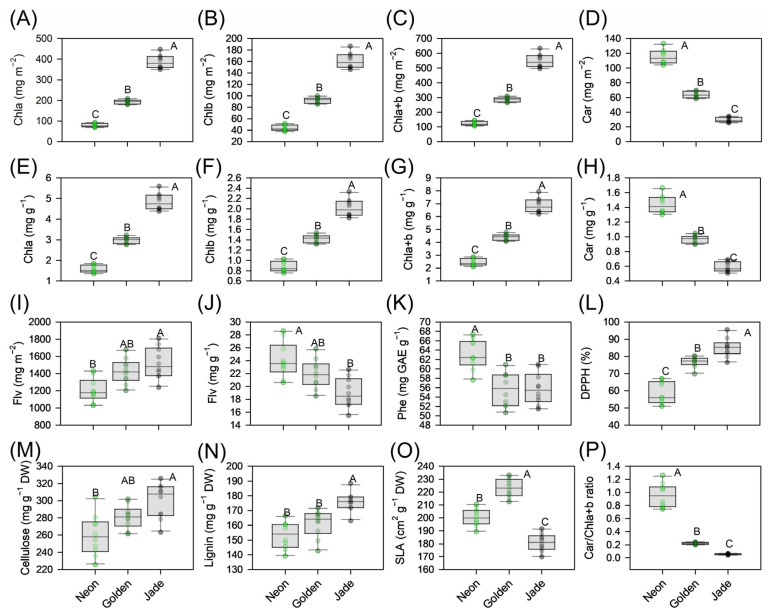
Leaf pigment, phenolic, antioxidant and structural traits of three leaf-colour phenotypes of *Epipremnum aureum* (‘Neon’, ‘Golden’ and ‘Jade’). (**A**–**D**) Area-based contents of chlorophyll a (Chla), chlorophyll b (Chlb), total chlorophyll (Chla+b) and carotenoids (Car) (mg m^−2^). (**E**–**H**) Mass-based contents of Chla, Chlb, Chla+b and Car (mg g^−1^ dry mass). (**I**,**J**) Area- and mass-based contents of flavonoids (Flv) (mg m^−2^ and mg g^−1^ dry mass). (**K**) Total soluble phenolics (Phe; mg gallic acid equivalents (GAE) g^−1^ dry mass). (**L**) DPPH radical-scavenging capacity (%). (**M**,**N**) Cellulose and lignin contents (mg g^−1^ dry mass). (**O**) Specific leaf area (SLA; cm^2^ g^−1^ dry mass). (**P**) Carotenoids-chlorophyll a + b ratio (Car/Chla+b ratio). Data are shown for ‘Neon’, ‘Golden’ and ‘Jade’ as indicated in the legend. Boxplots show the median, interquartile range and full range; symbols denote individual leaves (*n* = 10 per phenotype). Different uppercase letters denote significant differences among phenotypes (*p* < 0.05; Tukey’s test).

This phenotypes variation is accompanied by coordinated shifts in leaf construction and pigment patterns (Figure 2A–P). ‘Jade’ leaves are thick, with dense mesophyll and the lowest specific leaf area, whereas ‘Golden’ leaves are thinnest and display the highest specific leaf area (Figure 2O); ‘Neon’ is intermediate in thickness but combines relatively high specific leaf area with lower structural investment in cellulose (Figure 2M) and lignin (Figure 2N). Dry mass per unit area is therefore highest in ‘Jade’, lowest in ‘Golden’ and intermediate in ‘Neon’.

Chlorophyll content follows the visual gradient, whereas the contents of carotenoids and phenolics do not scale proportionally with the amount of chlorophyll (Figure 2A–H). On a mass basis, ‘Neon’ shows the highest carotenoid:chlorophyll ratio and the highest flavonoid and total phenolic contents relative to chlorophyll (Figure 2I–K). In contrast, ‘Jade’ concentrates chlorophyll and carotenoids per unit area but maintains comparatively lower carotenoid:chlorophyll and flavonoid:chlorophyll ratios, with ‘Golden’ generally intermediate. Despite having the highest phenolic concentration, ‘Neon’ displays the weakest free radical-scavenging capacity (DPPH) (Figure 2L), whereas ‘Jade’ exhibits the strongest antioxidant activity despite lower phenolic levels (Figure 2K).

### 3.2. Optical Fingerprints Report Internal Pigment Gradients and Mesophyll Structure

**Figure 3:**
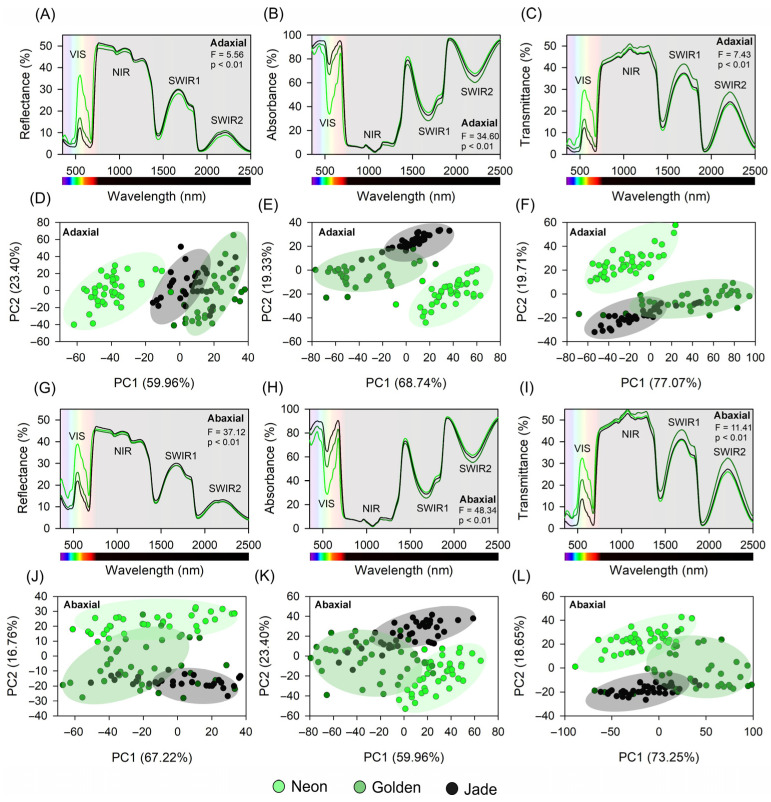
UV–VIS–NIR–SWIR hyperspectral leaf optical traits of three *Epipremnum aureum* leaf-colour phenotypes and their multivariate discrimination. (**A**–**C**) Adaxial reflectance, absorptance and transmittance spectra (350–2500 nm), with the visible (VIS), near-infrared (NIR) and short-wave infrared (SWIR1, SWIR2) regions indicated. (**D**–**F**) Principal component analysis (PCA) score plots of adaxial spectra derived from reflectance, absorptance and transmittance, respectively. (**G**–**I**) As in (**A**–**C**), but for the abaxial surface. (**J**–**L**) PCA score plots of abaxial spectra. Lines in spectral panels represent mean spectra for each phenotype; points in the PCA plots correspond to individual leaves and ellipses to the multivariate dispersion of each phenotype. F-values from one-way ANOVA for phenotype effects on spectral signatures are shown in the spectral panels (*p* < 0.01). Hyperspectral measurements were made on 40 leaves per phenotype (4 fully expanded leaves per plant; *n* = 10 plants per phenotype).

Hyperspectral reflectance and transmittance captured cultivar-specific optical fingerprints consistent with the biochemical and anatomical contrasts described above (Figure 3A–L). In the 350–700 nm region, ‘Neon’ reflected and transmitted high green–yellow wavelengths and showed weaker absorption in the blue and red bands than ‘Jade’, whereas ‘Golden’ was generally intermediate, consistent with mixed contributions from variegated sectors. In the near-infrared (NIR; ~700–1000 nm) and short-wave infrared (SWIR; ~1000–2500 nm) ranges, ‘Jade’ exhibited slightly higher reflectance and lower transmittance than ‘Neon’ and ‘Golden’, consistent with greater scattering in its thicker mesophyll and higher structural density.

Principal component analysis of adaxial spectra separated the phenotypes primarily along a component dominated by visible-band absorption and red-edge position. The ‘Neon’ grouped with weak chlorophyll absorption and strong green reflectance, ‘Jade’ with deep absorption and a pronounced red edge (700–1000 nm), and ‘Golden’ formed an intermediate cloud (Figure 3A–F). On the abaxial surface, separation was driven more by near-infrared scattering, mirroring differences in tissue thickness (Figure 3G–L). Together, these spectral patterns encode both pigment-related and cell structural variation among cultivars grown under identical irradiance.

### 3.3. Leaf Tissue Organisation and Stomatal Anatomical

**Figure 4:**
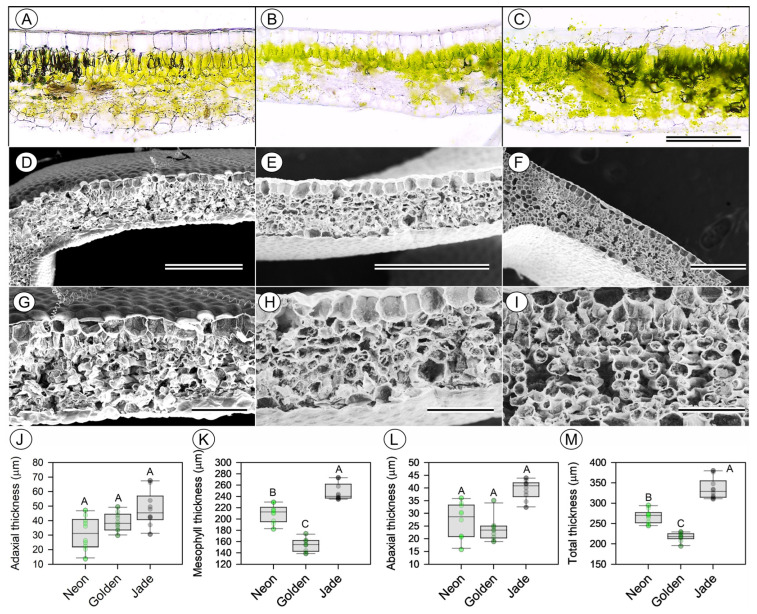
Leaf cross-sectional anatomy and thickness of three leaf-colour phenotypes of *Epipremnum aureum* (Linden & André) G.S. Bunting. (**A**–**C**) Bright-field transverse sections of fully expanded leaves from ‘Neon’, ‘Golden’ and ‘Jade’ plants. (**D**–**F**) Scanning electron microscopy (SEM) cross-sections at lower magnification showing overall lamina architecture. (**G**–**I**) Higher-magnification SEM cross-sections highlighting adaxial and abaxial epidermis and mesophyll organisation. (**J**–**M**) Thickness of the adaxial epidermis, mesophyll, abaxial epidermis and total leaf, respectively. Boxplots show the median, interquartile range and full range; the symbols denote individual leaves (*n* = 10 per phenotype). Different uppercase letters indicate significant differences among the phenotypes (*p* < 0.05; Tukey’s test). Scale bars = 300 µm (**A**–**C**), 500 µm (**D**–**F**) and 150 µm (**G**–**I**).

**Figure 5:**
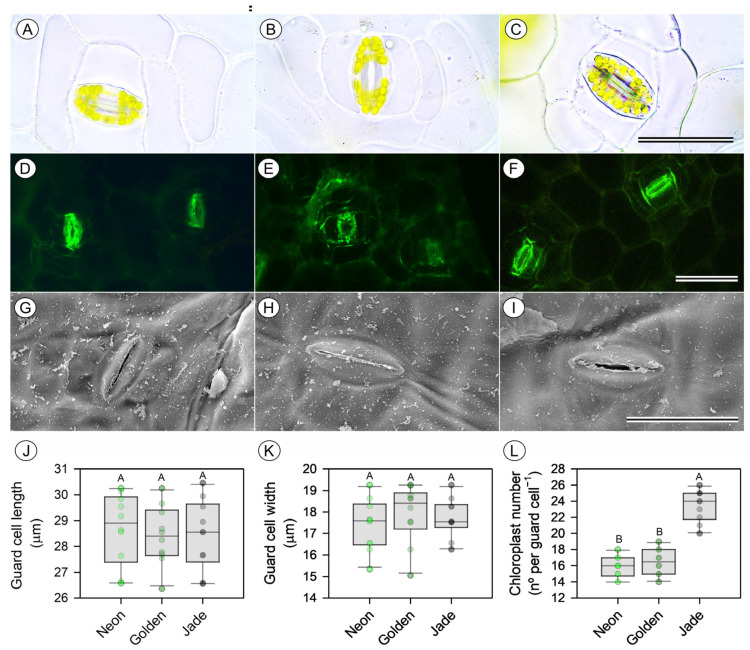
Stomatal structure and guard-cell chloroplast number in three leaf-colour phenotypes of *Epipremnum aureum* (Linden & André) G.S. Bunting. (**A**–**C**) Bright-field images of stomata showing the chloroplast distribution in guard cells of ‘Neon’, ‘Golden’ and ‘Jade’ leaves. (**D**–**F**) Corresponding epifluorescence images (fluorescein diacetate (FDA) signal) of guard cells. (**G**–**I**) Scanning electron micrographs of stomatal complexes on the leaf epidermis. (**J**,**K**) Guard-cell length and width. (**L**) Number of chloroplasts per guard cell. Boxplots show the median, interquartile range and full range; points represent individual stomata (*n* = 10 leaves per phenotype). Different uppercase letters denote significant differences among the phenotypes (*p* < 0.05; Tukey’s test). Scale bars = 50 µm (**A**–**I**).

Light microscopy and scanning electron microscopy confirm that cultivar differences in leaf construction arise primarily from the mesophyll (Figure 4 and Figure 5). ‘Jade’ leaves have the thickest palisade and spongy mesophyll, whereas ‘Golden’ leaves have a more compressed mesophyll with larger intercellular air spaces; ‘Neon’ leaves have intermediate palisade and spongy mesophyll (Figure 5A–J). Stomatal complexes are structurally similar among cultivars, but the stomatal density and stomatal index are greater on both leaf surfaces in ‘Neon’ and ‘Golden’ than in ‘Jade’, indicating a greater stomatal investment in pale and variegated leaves (Figure 4A–L).

### 3.4. Chloroplast Density and Thylakoid Architecture Diverge Strongly Among Cultivars

**Figure 6:**
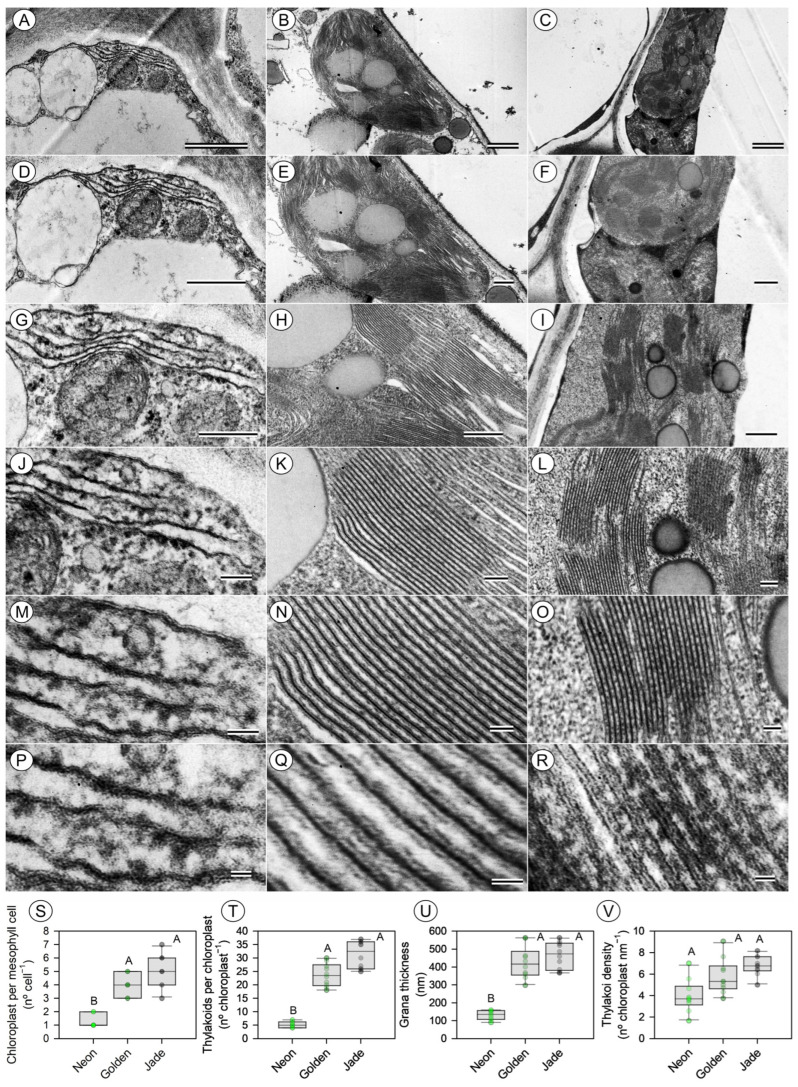
Chloroplast ultrastructure and thylakoid organisation in three leaf-colour phenotypes of *Epipremnum aureum* (Linden & André) G.S. Bunting. Transmission electron micrographs of palisade mesophyll chloroplasts from (**A**,**D**,**G**,**J**,**M**,**P**) ‘Neon’, (**B**,**E**,**H**,**K**,**N**,**Q**) ‘Golden’ and (**C**,**F**,**I**,**L**,**O**,**R**) ‘Jade’ leaves at increasing magnification, highlighting overall chloroplast shape, stromal volume and grana stacking. Scale bars (top to bottom rows) = Transmission electron microscopy (TEM) images primarily showing chloroplast structures. Scale bars (top to bottom rows): 1 μm (**A**–**C**); 500 nm (**D**–**F**); 400 nm (**G**–**I**); 100 nm (**J**–**L**); 50 nm (**M**–**O**); and 20 nm (**P**–**R**), as indicated in each panel. Quantitative ultrastructural traits: (**S**) number of chloroplasts per mesophyll cell, (**T**) number of thylakoids per chloroplast, (**U**) grana thickness and (**V**) thylakoid density per unit of chloroplast units. Boxplots show the median, interquartile range and full range; points represent individual chloroplasts or cells (*n* = 10 leaves per phenotype). Different uppercase letters denote significant differences among the phenotypes (*p* < 0.05; Tukey’s test). In Figure 6, the images relate mainly to chloroplasts, whereas in Figure 7 they relate to mitochondria, vesicles, other organelles, and the cell wall.

**Figure 7:**
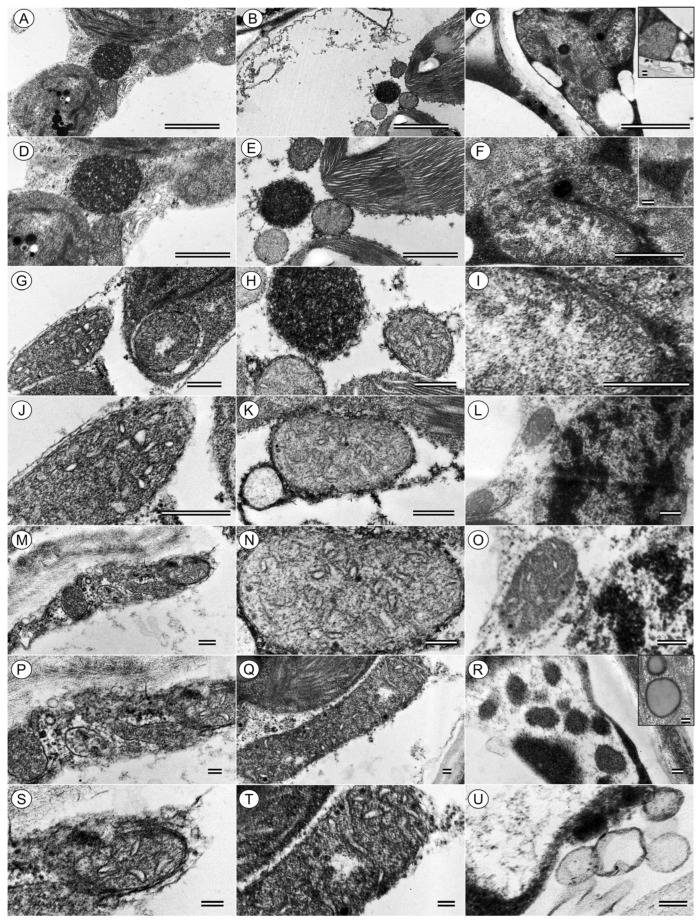
Transmission electron microscopy of chloroplasts and associated organelles in mesophyll cells of *Epipremnum aureum* leaves. Representative micrographs for (**A**,**D**,**G**,**J**,**M**,**P**,**S**) ‘Neon’, (**B**,**E**,**H**,**K**,**N**,**Q**,**T**) ‘Golden’ and (**C**,**F**,**I**,**L**,**O**,**R**,**U**) ‘Jade’ phenotypes illustrate the spatial association of chloroplasts with mitochondria, peroxisomes and vacuoles, together with higher-magnification views of chloroplast envelopes, thylakoid systems, plastoglobuli and organelle contact sites. Scale bars = Transmission electron microscopy (TEM) images showing mitochondria, vesicles, other organelles, and the cell wall. Scale bars: 2 μm (**A**,**B**); 500 nm (**C**,**G**–**I**); 1 μm (**D**,**E**); 200 nm (**F**,**M**–**O**); 400 nm (**J**–**L**); and 100 nm (**P**–**U**), as indicated in each panel. Insets: 100 nm (**C**,**F**,**R**). Overlay comparisons are shown between Figure 6F and Figure 7C–F and between Figure 6A and Figure 7M. Insets: panels (**C**,**F**,**R**) correspond to a scale bar of 100 nm.

Transmission electron microscopy reveals marked divergence in chloroplast abundance and thylakoid organisation (Figure 6, Figure 7 and Figure S1). ‘Jade’ mesophyll cells contain, on average, more than fourfold more chloroplasts than ‘Neon’ cells (Figure 6A,D,G,J,M,P), and each ‘Jade’ chloroplast contains numerous well-stacked thylakoid lamellae organised into thick grana (Figure 6B,E,H,K,N,Q). ‘Golden’ cells contain slightly fewer chloroplasts and thinner grana than ‘Jade’ (Figure 6C,F,I,L,O,R) but still far more than ‘Neon’ (Figure 6A–R). When chloroplast number and thylakoid number per chloroplast are combined, ‘Jade’ mesophyll contains approximately an order of magnitude more thylakoid stacks per cell than ‘Neon’ and several-fold more than ‘Golden’. By contrast, differences in thylakoid density per chloroplast area were smaller than the differences observed for chloroplast number per cell and thylakoid number per chloroplast. Thus, variegation appears to affect thylakoid abundance per cell more strongly than this area-normalised density metric (Figure 6S–V and Figure S1).

Variegated tissues in ‘Golden’ and the palest cells in ‘Neon’ frequently contain plastids with poorly developed thylakoids, expanded stroma and abundant plastoglobuli, whereas ‘Jade’ chloroplasts are more homogeneous and often contain minor or not clearly identified starch grains (Figure 6 and Figure 7). These ultrastructural features are consistent with a gradient from fully developed, energy-transducing chloroplasts (align quantification data, see Section 3.5) in ‘Jade’ to partially differentiated or degraded plastids in the palest ‘Neon’ tissues. Consistent with the higher-magnification survey (Figure 7), ‘Jade’ mesophyll cells also exhibit frequent chloroplast–mitochondria associations and relatively small plastoglobuli, whereas ‘Neon’ and pale ‘Golden’ sectors show more isolated plastids with enlarged plastoglobuli and fewer apparent organelle contacts.

### 3.5. PSII Units in Chlorophyll-Poor Leaves Work Harder and Dissipate More Energy per Reaction Centre

**Figure 8:**
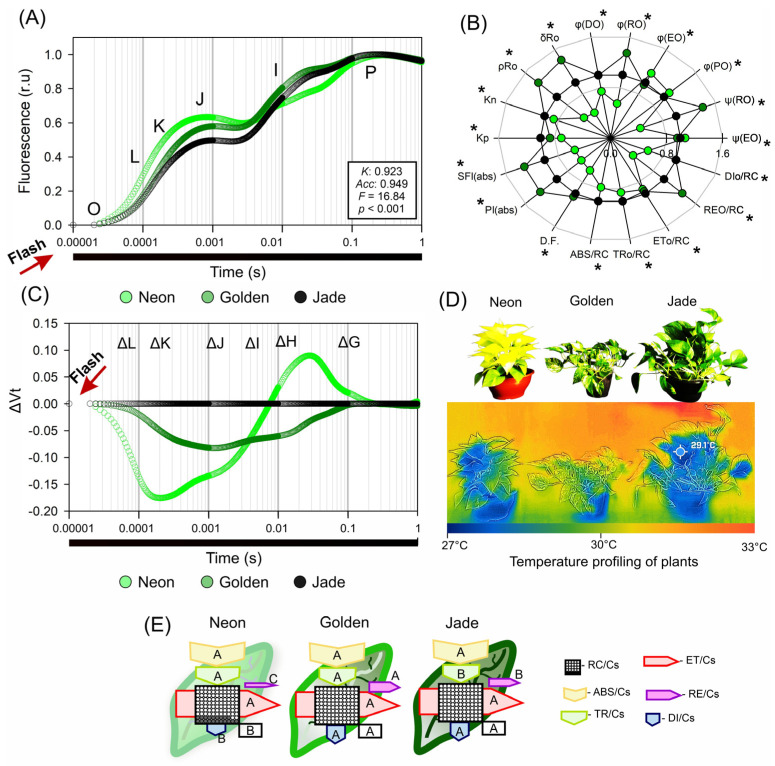
Chlorophyll a fluorescence induction kinetics and JIP-test energy-flux parameters in three *Epipremnum aureum* leaf-colour phenotypes. (**A**) Chlorophyll a fluorescence induction kinetics (OJIP transients; relative fluorescence, log time scale) for ‘Neon’, ‘Golden’ and ‘Jade’ leaves in the specific O, L, K, J, I, P-bands. (**B**) Radar plot of selected JIP-test parameters [ψ(EO), φ(EO), φ(PO), φ(RO), δR_0_, ρR_0_, Kp, Kn], summarising changes in PSII excitation energy trapping, electron transport and recombination among phenotypes; different uppercase asterisks on each axis denote significant differences (*p* < 0.001; Tukey’s test). (**C**) Relative difference kinetics (ΔV) highlighting cultivar-dependent deviations in the specific ΔL-, ΔK-, ΔJ-, ΔI-, ΔH- and ΔG-bands; in ‘Neon’, the most evident deviation occurs in the early OJIP phase and is consistent with donor-side perturbation. (**D**) False-colour thermal images of ‘Neon’, ‘Golden’ and ‘Jade’ plants acquired with an infrared camera, showing cultivar differences in canopy surface temperature under the same greenhouse conditions. Thermal imaging provides an integrative readout of leaf energy balance (including transpiration-driven cooling) and is presented alongside fluorescence-derived traits as a complementary phenotype. (**E**) Pipeline–leaf diagrams showing phenomenological energy flows per excited PSII cross-section (CS): yellow arrow, absorbed energy (ABS/CS); green arrow, trapped energy leading to QA reduction (TR/CS); red arrow, electron transport beyond QA^−^ (ET/CS); blue arrow, dissipated energy (DI/CS); squares with circles, fraction of QA-reducing reaction centres (RC/CS). Arrow and symbol sizes depict the redistribution of absorbed energy between photochemistry and non-photochemical dissipation among phenotypes. Different capital letters indicate statistically significant differences for the pipeline–diagrams in phenomenological energy fluxes. Values are means ± SE (*n* = 10 plants per phenotype).

The OJIP transients (Figure 8) showed that, despite strong structural contrasts among the cultivars, the maximum PSII quantum efficiency remained high, with only modest differences in JIP-test parameters (Figure 8A–E). Differences became evident when subtle deviations in the normalised fluorescence increase were resolved and expressed as JIP-test parameters (Figure 8B,C). Relative to ‘Jade’, ‘Neon’ displayed pronounced negative L- and K-band signatures, consistent with altered donor-side behaviour during the early OJIP phase (Figure 8C). ‘Neon’ also exhibited consistently higher specific energy fluxes per active reaction centre (ABS/RC, TR0/RC, ET0/RC and DI0/RC) than ‘Jade’ and ‘Golden’, with particularly elevated absorption and dissipation per reaction centre (Figure 8D,E). In parallel, ‘Neon’ showed higher probabilities and yields of electron transfer towards PSI terminal acceptors, reflected by increased ψR0, φR0, δR0 and ρR0 (Figure 8B–E).

Performance indices normalised per reaction centre were highest in ‘Neon’, despite its low chloroplast and thylakoid abundance per cell (Figure 8B,E). Accordingly, each remaining functional PSII unit in ‘Neon’ carried a higher excitation load and dissipated a greater share as heat, whereas ‘Jade’ distributed excitation across a dense population of reaction centres with more modest specific fluxes (Figure 8D,E). ‘Golden’ was generally intermediate, consistent with its mosaic ultrastructure (Figure 8E). Sector-specific functional differences were not quantified separately and are therefore interpreted qualitatively.

Across leaves, ABS/RC and DI0/RC correlated with the carotenoid concentration and phenolic content and even more strongly correlated with the fraction of closed reaction centres (1 − qL) and with spectral features in the green–yellow region (Figure 8E; RC/CSs). Together, these relationships place carotenoid-rich, chlorophyll-poor ‘Neon’ at the high end of the excitation pressure per active PSII, coupled with enhanced heat dissipation and elevated electron transfer towards PSI acceptors.

Thermal imaging revealed a clear cultivar gradient in canopy surface temperature (Figure 8D). ‘Neon’ exhibited the highest canopy temperature, whereas ‘Jade’ remained coolest, with ‘Golden’ intermediate. These differences reflect cultivar-specific leaf energy balance under the shared greenhouse environment and should be interpreted in conjunction with gas-exchange traits (g_s_ and E; Table 1) and optical/structural differences affecting radiation absorption and internal energy use. Therefore, we discuss canopy temperature as a complementary, integrative phenotype that co-varies with photosynthetic regulation, rather than as a direct outcome of PSII thermal dissipation.

### 3.6. Dynamic Fluorescence Links Thylakoid Architecture to Non-Photochemical Quenching and Water-Use Efficiency

**Figure 9:**
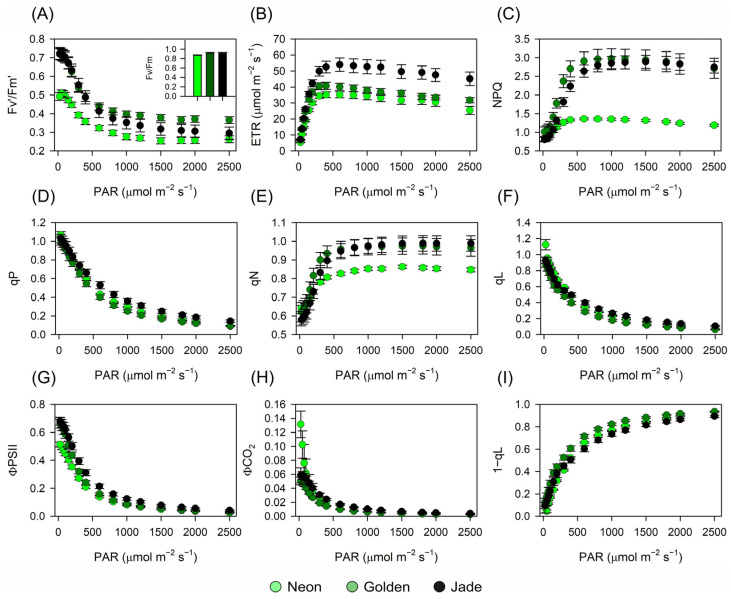
Chlorophyll a fluorescence-derived photochemical and non-photochemical responses to increasing photosynthetically active radiation (PAR) in three *Epipremnum aureum* leaf-colour phenotypes. (**A**) Maximum efficiency of PSII in the light-adapted state (Fv′/Fm′); inset, dark-adapted maximum PSII efficiency (Fv/Fm) >0.8 units. (**B**) Electron transport rate (ETR). (**C**) Non-photochemical quenching (NPQ). (**D**) Photochemical quenching coefficient (qP). (**E**) Non-photochemical quenching coefficient (qN). (**F**) Fraction of open PSII centres (qL). (**G**) Quantum yield of PSII photochemistry (ΦPSII). (**H**) Apparent quantum yield of CO_2_ assimilation (ΦCO_2_). (**I**) 1 − qL, estimating the reduction state of the PSII acceptor side. Curves are shown for ‘Neon’, ‘Golden’ and ‘Jade’ leaves. Symbols represent means ± SE (*n* = 10 plants per phenotype). Light–response curves differ among phenotypes, as determined by Tukey’s test (*p* < 0.01).

Non-steady-state chlorophyll fluorescence was measured under stepwise increases in actinic irradiance in separated cultivars primarily by operating PSII efficiency, electron transport capacity and energy dissipation (Figure 9 and Table 1). Maximum PSII efficiency (Fv/Fm) was similar across cultivars (Figure 9A, inset), but Fv′/Fm′ and ΦPSII declined more steeply with irradiance in ‘Neon’, whose ETR saturated at the lowest level (Figure 9A,B,G). By contrast, ‘Golden’ expressed the strongest dissipative response: NPQ (and qN) rose rapidly with PAR to values comparable to, and at some irradiances slightly exceeding, ‘Jade’, whereas ‘Neon’ maintained consistently low NPQ across the light gradient (Figure 9C,E). Photochemical quenching indices (qP and qL) decreased with irradiance in all cultivars, while the complementary excitation–pressure index (1 − qL) increased with PAR and remained cultivar-dependent (Figure 9D,F,I). Across the same irradiance series, ΦCO_2_ declined with PAR concomitantly with the increase in 1 − qL (Figure 9H,I).

### 3.7. Variegation Uncouples CO_2_ Diffusion, Biochemical Capacity and Carbon Storage

**Figure 10:**
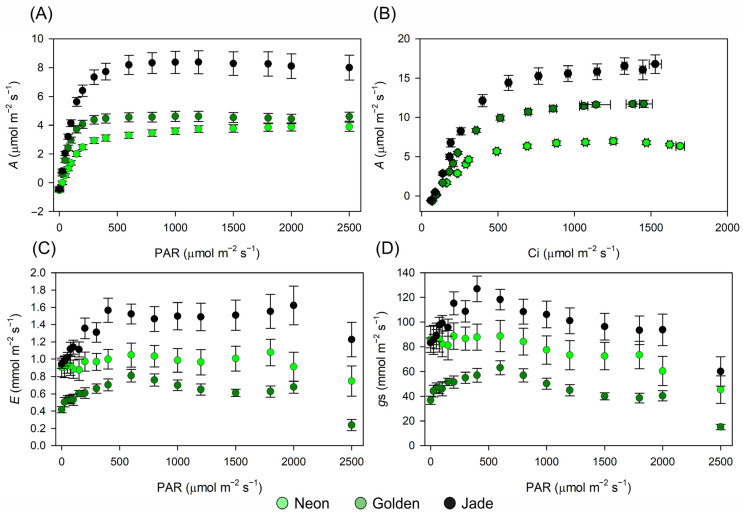
Photosynthetic and gas-exchange responses in three *Epipremnum aureum* leaf-colour phenotypes. (**A**) Light–response curves of net CO_2_ assimilation rate (**A**) as a function of photosynthetically active radiation (PAR; µmol m^−2^ s^−1^). (**B**) A–C_i_ curves of A versus intercellular CO_2_ concentration (C_i_; µmol mol^−1^). (**C**) Light–response curves of transpiration rate (E; mmol m^−2^ s^−1^). (**D**) Light–response curves of stomatal conductance to water vapour (g_s_; mmol m^−2^ s^−1^). Curves are shown for ‘Neon’, ‘Golden’ and ‘Jade’ leaves. Symbols represent means ± SE (*n* = 10 plants per phenotype).

Gas-exchange responses diverged and strongly differed among the cultivars across both the light and CO_2_ gradients (Figure 10; Table 1). In the A–PAR response, ‘Jade’ reached the highest light-saturated net CO_2_ assimilation and showed the highest apparent quantum yield, whereas ‘Neon’ exhibited the weakest light response, with a markedly greater light compensation point and the lowest maximum assimilation. ‘Golden’ had an intermediate in maximum A but reached light saturation at substantially lower irradiance than ‘Neon’ and ‘Jade’ (Figure 10A; Table 1).

The A–C_i_ curves and fitted parameters showed that the carboxylative capacity was high in ‘Golden’ and ‘Jade’ but substantially reduced in ‘Neon’, while RuBP-regeneration capacity and TPU were highest in ‘Jade’, intermediate in ‘Golden’ and lowest in ‘Neon’ (Figure 10B; Table 1). Consistent with these patterns, both the A–PAR-derived ATP/NADPH equivalents and the A–C_i_ model-derived ATP/NADPH equivalents associated with J_MAX_ scaled with cultivar photosynthetic capacity (Table 1). Because these quantities originate from different analytical contexts, they were interpreted as comparative, model-derived equivalents rather than as a strict supply–demand closure. At saturating irradiance, quantum yields were also cultivar-dependent: ΦPSII was highest in ‘Jade’, intermediate in ‘Golden’ and lowest in ‘Neon’, whereas ΦCO_2_ was highest in ‘Jade’ and lowest in ‘Golden’ (Table 1).

**Figure 11:**
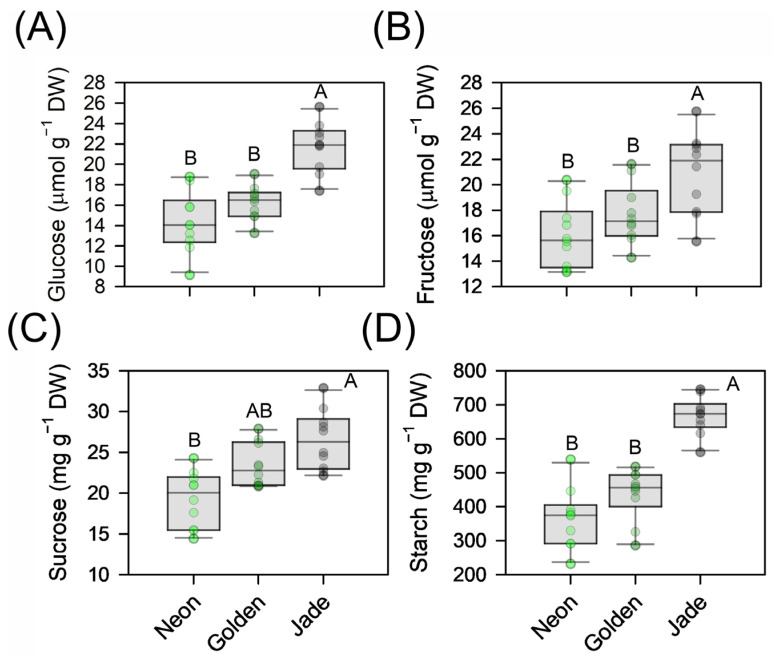
Soluble sugar and starch pools in leaves of three *Epipremnum aureum* colour phenotypes. (**A**–**C**) Contents of glucose, fructose (µmol g^−1^ dry mass) and sucrose (mg g^−1^ dry mass) in fully expanded leaves of ‘Neon’, ‘Golden’ and ‘Jade’ plants. (**D**) Starch content (mg g^−1^ dry mass) determined from the same samples. Boxplots represent the distribution of leaf-level carbohydrate pools within each phenotype; points denote individual leaves (*n* = 10 per phenotype). Different uppercase letters indicate significant differences among the phenotypes (*p* < 0.05; Tukey’s test).

Diffusive behaviour did not scale with biochemical capacity. Across the irradiance series, ‘Golden’ maintained lower stomatal conductance and transpiration than ‘Neon’ and ‘Jade’ (Figure 10C,D), yet it achieved the highest intrinsic water-use efficiency, whereas ‘Neon’ was least efficient (Table 1). Carbohydrate pools mirrored these differences in carbon balance: ‘Jade’ accumulated higher glucose and fructose and stored substantially more starch than ‘Neon’ and ‘Golden’, whereas the amount of sucrose was intermediate in ‘Golden’ between the two extremes (Figure 11A–D).

### 3.8. Multivariate Integration and Latent Axis of Photon Economy

**Figure 12:**
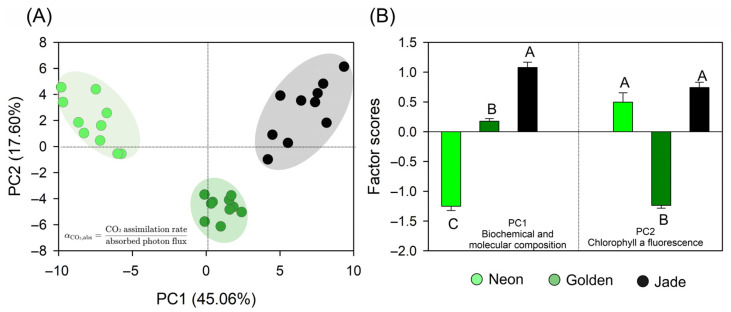
Multivariate integration of biochemical, structural and photosynthetic traits in *Epipremnum aureum* leaves explaining variation in photosynthetic quantum yield (αCO_2,abs_). (**A**) Principal component analysis (PCA) score plot for the ‘Neon’, ‘Golden’ and ‘Jade’ phenotypes; each point represents an individual leaf (*n* = 10), and the shaded ellipses indicate group dispersion. (**B**) Mean factor scores (means ± SE) for PC1 and PC2 for each phenotype, summarising coordinated shifts in biochemical and structural traits and in energy partitioning that underpin differences in αCO_2,abs_. Different uppercase letters above the bars indicate significant differences among the phenotypes for each principal component (*p* < 0.001; Tukey’s test).

Principal component analysis integrating all the trait groups separated the three cultivars into distinct, non-overlapping clusters (Figure 12A). PC1 explained 45.06% of the variance and defined the PCA photon-economy axis (αCO_2,abs_), positioning ‘Neon’ at low scores, ‘Jade’ at high scores and ‘Golden’ in between (Figure 12A,B).

Partitioning the explained variance by variable groups showed that PC1 was dominated by biochemical and molecular composition, with additional contributions from JIP-derived descriptors and phenomenological fluxes, whereas structure/ultrastructure and fluorescence-based PSII energy partitioning contributed comparatively less to this axis (Table 2). PC2 explained 17.60% of the variance and primarily separated ‘Golden’ from ‘Neon’ and ‘Jade’ (Figure 12A,B); consistent with the explanatory breakdown, this component was weighted most strongly by chlorophyll fluorescence-based PSII energy partitioning, together with A–PAR photochemical variables and JIP parameters (Table 2).

**Figure 13:**
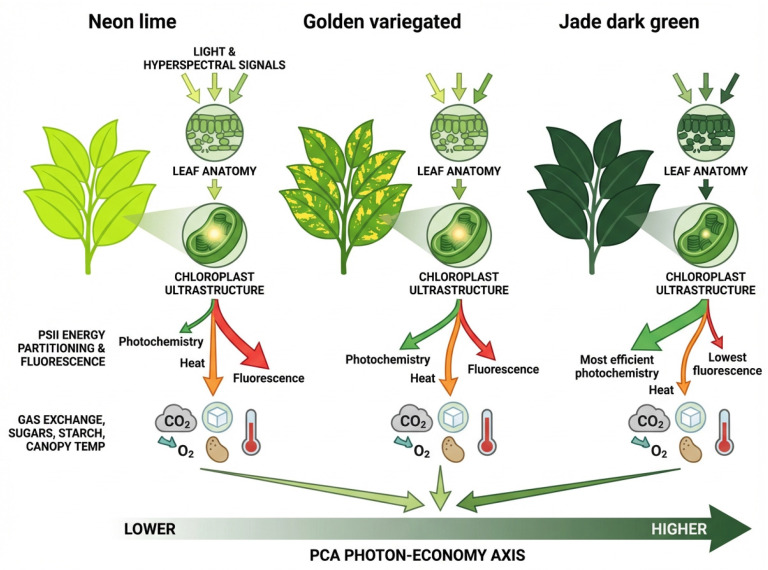
Conceptual model summarising the structural and functional determinants of photosynthetic quantum yield in three *Epipremnum aureum* (Linden & André) G.S. Bunting leaf-colour phenotypes (‘Neon’, ‘Golden’ and ‘Jade’). The scheme integrates differences in pigment composition and leaf optical properties, lamina anatomy and chloroplast ultrastructure, stomatal behaviour, chlorophyll a fluorescence-derived energy partitioning, gas exchange, carbohydrate pools, and leaf temperature and illustrates how these traits position the three phenotypes along a PCA-derived photon-economy axis from low (‘Neon’) to high (‘Jade’). Created in BioRender. Falcioni, R. (2026) https://BioRender.com/hcamxmo (accessed on 10 March 2026).

This multivariate structure underpins the conceptual synthesis in Figure 13, which places the three phenotypes along a continuum of photon economy emerging from coordinated variation across optics, anatomy, chloroplast ultrastructure and carbon gain.

## 4. Discussion

The three *Epipremnum aureum* phenotypes show that variegation involves not only pigment dilution but also coordinated variation across mesophyll construction, chloroplast deployment and thylakoid architecture that propagates to whole-leaf function, which is consistent with organelle-centred frameworks emphasising plastid developmental state and inter-organelle coupling as key constraints on performance [16,17,18]. By redistributing chloroplasts and thylakoid stacks among mesophyll cells, the cultivars reallocate the excitation load across PSII units and reshape investment in structural and biochemical machinery per unit of leaf area (Figure 13). This finding supports H1, in which variegation reshapes the internal photon field through coupled changes in absorption and scattering across the lamina [4,6,17,18].

‘Jade’ exemplifies a high-chloroplast-density strategy. Thick mesophyll and abundant chloroplasts bearing thick grana increase reaction-centre density and support high area-based photochemical throughput and CO_2_-fixation capacity, which is consistent with the combination of high ΦPSII, high Vc_MAX_/J_MAX_/TPU and substantial carbohydrate accumulation [24,25]. This configuration also sustains robust dissipation capacity and cooler canopies, consistent with more effective energy-balance regulation, including transpiration-driven cooling [45,46,52,53].

‘Neon’ represents the opposite extreme, a low chloroplast-density phenotype in which absorbed energy is concentrated onto fewer functional PSII units. Elevated specific fluxes per reaction centre and signatures of higher excitation pressure indicate that the remaining PSII units operate closer to their dissipative limits, a pattern consistent with systems where reaction-centre density per area is reduced [29,42,43]. Although ‘Neon’ shows high carotenoid- and phenolic-to-chlorophyll ratios, its operating photochemical efficiency declines rapidly with irradiance and its carbon economy remains constrained, with reduced carboxylative and RuBP-regeneration capacities, a high light compensation point and small carbohydrate reserves. High stomatal conductance does not translate into proportionate carbon gain, consistent with low intrinsic water-use efficiency and warmer canopies [53,54,55].

‘Golden’ does not simply sit between ‘Jade’ and ‘Neon’; it expresses a distinct mosaic design. Despite a thin mesophyll and early light saturation, ‘Golden’ maintains high biochemical capacity while operating with more conservative stomatal conductance, resulting in high intrinsic water-use efficiency [56,57,58,59,60]. The PSII units in ‘Golden’ express strong NPQ and high qN, and leaves reach light saturation at relatively low irradiance, limiting the time spent under severe excitation pressure, in line with recent analysis of dynamic NPQ components and their deployment across light regimes [53,54]. Sectoral organisation is consistent with the coexistence of well-developed photosynthetic tissue alongside pale sectors with impaired plastid development, providing a mechanistic precedent for partial disruption of tetrapyrrole-linked chloroplast biogenesis in variegated tissues. Classical Arabidopsis genomes uncoupled mutants in *GUN1* and *GUN4*, which respectively tune biogenic plastid-to-nucleus signalling and Mg-chelatase-dependent tetrapyrrole flux, and water-use efficiency illustrates that perturbations in these modules generate chlorophyll-poor, developmentally arrested chloroplasts with impaired thylakoid architecture [2,3,10], a syndrome that visually resembles the stable pale and variegated sectors of *E. aureum* ‘Neon’ and ‘Golden’ and therefore provides a mechanistic framework for hypothesising local defects in tetrapyrrole-mediated chloroplast biogenesis in these cultivars.

Together, these phenotypes show how variegation and chlorophyll dilution can remain physiologically viable without catastrophic loss of function. In line with H2, chlorophyll-poor leaves accommodate reduced photosynthetic machinery by increasing reaction-centre workload and shifting energy partitioning towards dissipation and distal electron flow, but net carbon gain remains limited by biochemical capacity [2,4,5]. In line with H3, these constraints cascade to diffusive control and water economy, coinciding with distinct canopy thermal regimes as an integrative outcome of the leaf–energy balance [16,18].

The strong association between NPQ capacity and thylakoid architecture is particularly informative. This implies that the grana scaffold and the abundance of pigment–protein complexes impose a structural ceiling on the maximum thermal dissipation achievable at the tissue level, whereas the carotenoid composition modulates the dissipative propensity per complex [15,54]. This framework explains why ‘Neon’, despite very high carotenoid:chlorophyll ratios, expresses only modest NPQ: with few thylakoids per cell [53,61], it lacks a sufficient dissipative ‘surface area’ to sustain large thermal quenching. By contrast, ‘Golden’ and ‘Jade’, with many more thylakoids, maintain high NPQ even at lower carotenoid ratios, which is consistent with recent syntheses of NPQ kinetics and antenna–membrane organisation [15,62,63].

The chloroplast–mitochondria contact frequency and plastoglobuli size along the variegation gradient (Figure 7) suggest that organelle interactions and thylakoid lipid remodelling are integral components of this structural control of photon economy. This finding dovetails with recent systems-level work showing that plastids behave as dynamic metabolic hubs whose functions are tightly integrated with those of mitochondria and other organelles [17,18]. Dynamic proteomic and lipidomic profiling of plastoglobules in maize further supports a role for plastoglobuli proliferation and compositional shifts in buffering thylakoid function during drought and heat cycles [64,65,66,67,68], which is consistent with earlier proposals that plastoglobuli act as microcompartments coordinating lipid metabolism, plastid developmental transitions and environmental adaptation.

Variation in cell-wall composition (cellulose and lignin) may also contribute to the optical and thermal phenotypes observed here. Differences in cell-wall density and fibre composition can alter mesophyll light scattering and internal path length, potentially modulating leaf absorptance and the spectral signatures in the NIR/SWIR bands. In addition, cell-wall traits can influence leaf mechanical properties and hydraulic function, indirectly affecting stomatal conductance and evaporative cooling (Figure 13). Finally, soluble sugars and starch pools provide an integrative readout of cumulative carbon gain, and their cultivar differences are consistent with the αCO_2,abs_ gradient and with the contrasting biochemical capacities inferred from A–C_i_ modelling.

In summary, the three *E. aureum* cultivars reveal variegation as a controllable reallocation between chloroplast number and reaction centre workload that cascades through pigment pattern, thylakoid architecture, PSII energy partitioning, gas exchange, water-use efficiency and canopy temperature. By combining hyperspectral sensing, fluorescence kinetics and ultrastructural quantification, this work transforms variegated pothos from a decorative curiosity into a mechanistic model for how leaves restructure their photon economy to cope with low irradiance, as encountered in indoor and shaded greenhouse conditions, and complements recent frameworks that link spectral traits and NPQ dynamics to photosynthetic capacity and resilience [19,20,24,38].

Collectively, ‘Neon’ and ‘Jade’ illustrate contrasting strategies: ‘Neon’ operates with few, chlorophyll-poor chloroplasts and high excitation load per PSII centre, relying on elevated carotenoid ratios that nonetheless do not restore αCO_2,abs_; ‘Jade’ achieves high photon economy through dense chloroplast populations and extensive grana that distribute excitation and sustain both high carbon gain and cooler canopies.

## 5. Conclusions

Our results demonstrate that, across the examined variegated *Epipremnum aureum*, photosynthetic quantum yield and net carbon gain are more strongly associated with mesophyll-scale chloroplast deployment and thylakoid/grana organisation than with carotenoid enrichment of the pigment pool. Differences in thylakoid membrane abundance altered PSII reaction-centre density and excitation pressure, thereby constraining the capacity for regulated thermal energy dissipation and the operating efficiency of electron transport. These photochemical constraints propagated to the carboxylative phase by coordinating ATP and NADPH supply with Rubisco carboxylation, RuBP regeneration and TPU, ultimately shaping carbohydrate accumulation and intrinsic water-use efficiency, and co-varying with canopy temperature as an integrative outcome of leaf energy balance. A dominant multivariate axis of photon economy (αCO_2,abs_) integrated optical traits, chloroplast ultrastructure and gas exchange, providing a mechanistic basis for predicting when variegated leaves remain carbon-positive under low irradiance close to the CO_2_ compensation point.

## Figures and Tables

**Table 1:** Photochemical, carboxylative and PSII energy-partitioning parameters derived from A–PAR, A–C_i_ and chlorophyll a fluorescence in fully expanded leaves of three *Epipremnum aureum* (Linden & André) G.S. Bunting colour phenotypes (‘Neon’, ‘Golden’ and ‘Jade’). Values are means ± SE (*n* = 10). Different uppercase letters within a row indicate significant differences among phenotypes (Tukey’s test, *p* < 0.001). Photochemical variables from light response A–PAR curves: Rd, dark respiration (µmol CO_2_ m^−2^ s^−1^); LCP, light compensation point (µmol photons m^−2^ s^−1^); LSP, light saturation point (µmol photons m^−2^ s^−1^); PN_MAX_, maximum net CO_2_ assimilation at saturating PAR (µmol CO_2_ m^−2^ s^−1^); A_MAX_, net CO_2_ assimilation at 1200 µmol photons m^−2^ s^−1^ (µmol CO_2_ m^−2^ s^−1^); α, apparent quantum yield of CO_2_ assimilation per absorbed photon (mol CO_2_ mol^−1^ photons); ATP, ATP production rate (µmol ATP m^−2^ s^−1^); NADPH, NADPH production rate (µmol NADPH m^−2^ s^−1^); i*WUE*, intrinsic water-use efficiency (µmol CO_2_ mol^−1^ H_2_O). Carboxylative CO_2_ assimilation from A–C_i_: Rd*CO_2_, day respiration estimated from A–C_i_ (µmol CO_2_ m^−2^ s^−1^); Vc_MAX_, maximum Rubisco carboxylation rate (µmol CO_2_ m^−2^ s^−1^); TPU, triose-phosphate utilisation capacity (µmol m^−2^ s^−1^); J_MAX_, maximum electron transport rate supporting RuBP regeneration (µmol e^−^ m^−2^ s^−1^); g_s_, stomatal conductance to water vapour (mmol H_2_O m^−2^ s^−1^); g_m_, mesophyll conductance to CO_2_ (mmol m^−2^ s^−1^); Cc, chloroplastic CO_2_ concentration (mmol mol^−1^); Γ, net CO_2_ compensation point (µmol mol^−1^); Ci_SAT_, intercellular CO_2_ concentration at CO_2_-saturated photosynthesis (µmol mol^−1^); ATP_CO2_, ATP requirement for CO_2_ assimilation (µmol ATP m^−2^ s^−1^); NADPH_CO2_, NADPH requirement for CO_2_ assimilation (µmol NADPH m^−2^ s^−1^). Chlorophyll a fluorescence—PSII energy partitioning at 1200 µmol photons m^−2^ s^−1^: Fv′/Fm′, maximum PSII efficiency in the light-adapted state (dimensionless); ETR, electron transport rate (µmol e^−^ m^−2^ s^−1^); NPQ, non-photochemical quenching (dimensionless); qP, photochemical quenching coefficient (dimensionless); qN, non-photochemical quenching coefficient (dimensionless); qL, fraction of open PSII centres (dimensionless); ΦPSII, quantum yield of PSII photochemistry (dimensionless); ΦCO_2_, quantum yield of CO_2_ assimilation per incident photon (mol CO_2_ mol^−1^ photons); 1 − qL, fraction of closed PSII centres (dimensionless).

Parameters		‘Neon’	‘Golden’	‘Jade’
**Photochemical variables (A–PAR)**	Rd	0.58 ± 0.13 C	0.38 ± 0.10 B	0.45 ± 0.11 A
	LCP	20.69 ± 2.29 A	9.91 ± 3.17 B	8.81 ± 2.35 B
	LSP	407.01 ± 36.42 A	200.89 ± 30.99 B	391.33 ± 37.97 A
	PN_MAX_	3.96 ± 0.28 C	4.78 ± 0.31 B	8.63 ± 0.69 A
	A_MAX_	4.58 ± 0.27 C	5.01 ± 0.28 B	9.00 ± 0.77 A
	α	0.026 ± 0.001 C	0.043 ± 0.003 B	0.053 ± 0.002 A
	ATP	13.49 ± 0.69 C	15.35 ± 0.80 B	20.46 ± 1.44 A
	NADPH	8.99 ± 0.46 C	10.23 ± 0.53 B	13.64 ± 0.96 A
	iWUE	50.86 ± 4.86 C	93.97 ± 3.72 A	82.30 ± 5.34 B
**Carboxylative CO_2_ assimilation (A–C_i_)**	Rd*_CO2_	1.31 ± 0.10 C	2.72 ± 0.32 A	1.74 ± 0.21 B
	Vc_MAX_	22.11 ± 1.21 B	45.55 ± 6.24 A	45.67 ± 3.13 A
	TPU	3.57 ± 0.77 C	4.96 ± 0.15 B	12.21 ± 3.53 A
	J_MAX_	38.00 ± 1.87 C	67.03 ± 2.76 B	79.54 ± 4.56 A
	g_s_	86.13 ± 9.09 A	45.61 ± 3.39 B	83.69 ± 5.85 A
	g_m_	385.80 ± 126.57 B	354.82 ± 113.76 B	648.92 ± 66.27 A
	Cc	213.73 ± 12.42 A	175.66 ± 10.54 B	178.37 ± 10.95 B
	Γ	91.44 ± 4.25 A	92.71 ± 2.97 A	73.62 ± 3.51 B
	C_iSAT_	658.06 ± 47.04 B	696.28 ± 55.51 B	793.40 ± 24.74 A
	ATP_CO2_	14.25 ± 0.70 C	25.14 ± 1.03 B	29.83 ± 1.71 A
	NADPH_CO2_	9.50 ± 0.47 C	16.76 ± 0.69 B	19.88 ± 1.14 A
**Chlorophyll a fluorescence—PSII energy partitioning at 1200 µmol photons m^−2^ s^−1^**	Fv′/Fm′	0.28 ± 0.01 C	0.40 ± 0.02 A	0.35 ± 0.02 B
	ETR	34.38 ± 2.23 C	37.98 ± 1.91 B	52.66 ± 4.19 A
	NPQ	1.35 ± 0.03 B	2.97 ± 0.25 A	2.85 ± 0.17 A
	qP	0.296 ± 0.005 B	0.254 ± 0.014 C	0.352 ± 0.023 A
	qN	0.85 ± 0.01 B	0.97 ± 0.04 A	0.97 ± 0.04 A
	qL	0.232 ± 0.004 B	0.178 ± 0.011 C	0.265 ± 0.020 A
	ΦPSII	0.082 ± 0.003 C	0.090 ± 0.004 B	0.125 ± 0.010 A
	ΦCO_2_	0.0088 ± 0.0019 B	0.0060 ± 0.0003 C	0.0104 ± 0.0009 A
	1 − qL	0.768 ± 0.004 B	0.822 ± 0.011 A	0.735 ± 0.020 C

**Table 2:** Relative contribution of functional trait groups to the first two principal components (PC1 and PC2) from the principal component analysis integrating leaf traits associated with the photosynthetic quantum yield αCO_2,abs_ (CO_2_ assimilation rate per absorbed photon) in *Epipremnum aureum* (Linden & André) G.S. Bunting leaves. The table shows the percentage contributions of biochemical and molecular composition, structure and ultrastructure, photochemical variables from A–PAR curves, carboxylative CO_2_ assimilation from A–C_i_ curves, chlorophyll a fluorescence-derived PSII energy partitioning under high PAR, JIP-test parameters, and phenomenological energy fluxes per PSII cross-section. For each principal component, the contributions of all trait groups sum to 100%.

Groups	PC1 (%)	PC2 (%)
Biochemical and molecular composition	25.44	6.18
Structure and ultrastructure	11.42	14.31
Photochemical variables (A-PAR)	11.68	17.09
Carboxylative CO_2_ assimilation	11.00	10.41
Chlorophyll a fluorescence—PSII energy partitioning	7.38	22.22
JIP-parameters	16.80	17.41
Phenomenological fluxes	16.27	12.38
**TOTAL**	**100**	**100**

## Data Availability

The original contributions presented in this study are included in the article/Supplementary Materials. Further inquiries can be directed to the corresponding author.

## Supplementary Materials

The following supporting information can be downloaded at: https://www.mdpi.com/article/10.3390/cells15060514/s1, Figure S1: Transmission electron microscopy of chloroplasts in mesophyll cells of *Epipremnum aureum* leaves. Representative micrographs for (**A**,**D**,**G**,**J**,**M**) ‘Neon’, (**B**,**E**,**H**,**K**,**N**) ‘Golden’ and (**C**,**F**,**I**,**L**,**O**) ‘Jade’ phenotypes illustrate the general organisation of mesophyll cells and the spatial distribution of chloroplasts, together with progressively higher-magnification views of chloroplast morphology, thylakoid systems, grana stacking and membrane appression. Scale bars = 2 µm (**A**,**B**,**E**), 1 µm (**C**,**H**,**K**), 500 nm (**D**,**F**,**I**), 200 nm (**G**,**L**), 100 nm (**J**,**N**,**O**) and 50 nm (**M**), as indicated in each panel.

## Abbreviations

A, net CO_2_ assimilation rate; A_λ_, spectral absorptance; A–C_i_, CO_2_-response curve of A versus intercellular CO_2_ concentration; A–PAR, light–response curve of A versus photosynthetically active radiation; ABS/RC, absorbed energy flux per PSII reaction centre; ABS/CS, absorbed energy flux per PSII cross-section; A_MAX_, net CO_2_ assimilation at 1200 µmol photons m^−2^ s^−1^; ANOVA, analysis of variance; ATP_CO2_, ATP requirement for CO_2_ assimilation; ATP, adenosine triphosphate; Cc, chloroplastic CO_2_ concentration; Chl, chlorophyll; ChlF, chlorophyll a fluorescence; DI0/RC, dissipated energy flux per PSII reaction centre; DI0/CS, dissipated energy flux per PSII cross-section; DPPH, 2,2-diphenyl-1-picrylhydrazyl; E, transpiration rate; ETR, electron transport rate; F0, minimum fluorescence (dark-adapted); Fm, maximum fluorescence (dark-adapted); Fv/Fm, maximum PSII efficiency (dark-adapted); Fv′/Fm′, maximum PSII efficiency (light-adapted); FvCB, Farquhar–von Caemmerer–Berry model; GAE, gallic acid equivalents; *GLK*, *‘GOLDEN’2-LIKE*; g_m_, mesophyll conductance to CO_2_; g_s_, stomatal conductance to water vapour; iWUE, intrinsic water-use efficiency; JIP-test, analysis of OJIP fluorescence transients; J_MAX_, maximum electron transport capacity supporting RuBP regeneration; LCP, light compensation point; LSP, light saturation point; NADPH, nicotinamide adenine dinucleotide phosphate (reduced form); NADPH_CO_2__, NADPH requirement for CO_2_ assimilation; NIR, near-infrared; NPQ, non-photochemical quenching; OJIP, polyphasic chlorophyll fluorescence transient (O–J–I–P); OM, optical microscopy; PCA, principal component analysis; PC1/PC2, principal components 1 and 2; PFCW, protein-free cell wall; PN_MAX_, maximum net CO_2_ assimilation at saturating PAR; PPFD, photosynthetic photon flux density; PSI/PSII, photosystem I/II; qL, fraction of open PSII centres; qN, non-photochemical quenching coefficient; qP, photochemical quenching coefficient; QA, primary quinone electron acceptor of PSII; R_λ_, spectral reflectance; Rd, dark respiration; Rd*CO_2_, day respiration estimated from A–C_i_; RC, reaction centre; RuBP, ribulose-1,5-bisphosphate; SEM, scanning electron microscopy; SLA, specific leaf area; SWIR, short-wave infrared; T_λ_, transmittance; TEM, transmission electron microscopy; TPU, triose-phosphate utilisation; UV–VIS, ultraviolet–visible; Vc_MAX_, maximum Rubisco carboxylation rate; VIS, visible; ΦCO_2_, quantum yield of CO_2_ assimilation per incident photon; ΦPSII, quantum yield of PSII photochemistry; α, apparent quantum yield from A–PAR; αCO_2,abs_, quantum yield of CO_2_ assimilation per absorbed photon; Γ, CO_2_ compensation point.
