# Supplementary material for: Chloroplast–Thylakoid Organisation Is More Important than Carotenoid Accumulation for Optimum Photosynthetic Quantum Yield and Carbon Gain in Variegated Epipremnum aureum
Source: Cells. 2026 Mar 13;15(6):514. doi: 10.3390/cells15060514 (PMC13026040; doi:10.3390/cells15060514)
Supplement: Supplementary file 1 [file cells-15-00514-s001.zip › cells-4178179-supplementary.pdf]

cells

MDPI

Supplementary file

Chloroplast–Thylakoid Organisation Is More Important than Carotenoid Accumulation for Optimum Photosynthetic Quantum Yield and Carbon Gain in Variegated *Epipremnum aureum*

Renan Falcioni [1,*], Werner Camargos Antunes [1], Marcelo Luiz Chicati [1], José Alexandre M. Demattê [2] and Marcos Rafael Nanni [1]

[1] Graduate Program in Agronomy, State University of Maringá, Av. Colombo, 5790, Maringá 87020-900, PR, Brazil; wcantunes@uem.br (W.C.A.); mlchicati@uem.br (M.L.C.); mrnanni@uem.br (M.R.N.)
[2] Department of Soil Science, Luiz de Queiroz College of Agriculture, University of São Paulo, Av. Pádua Dias, 11, Piracicaba 13418-260, SP, Brazil; jamdemat@usp.br
* Correspondence: rfalcioni2@uem.br

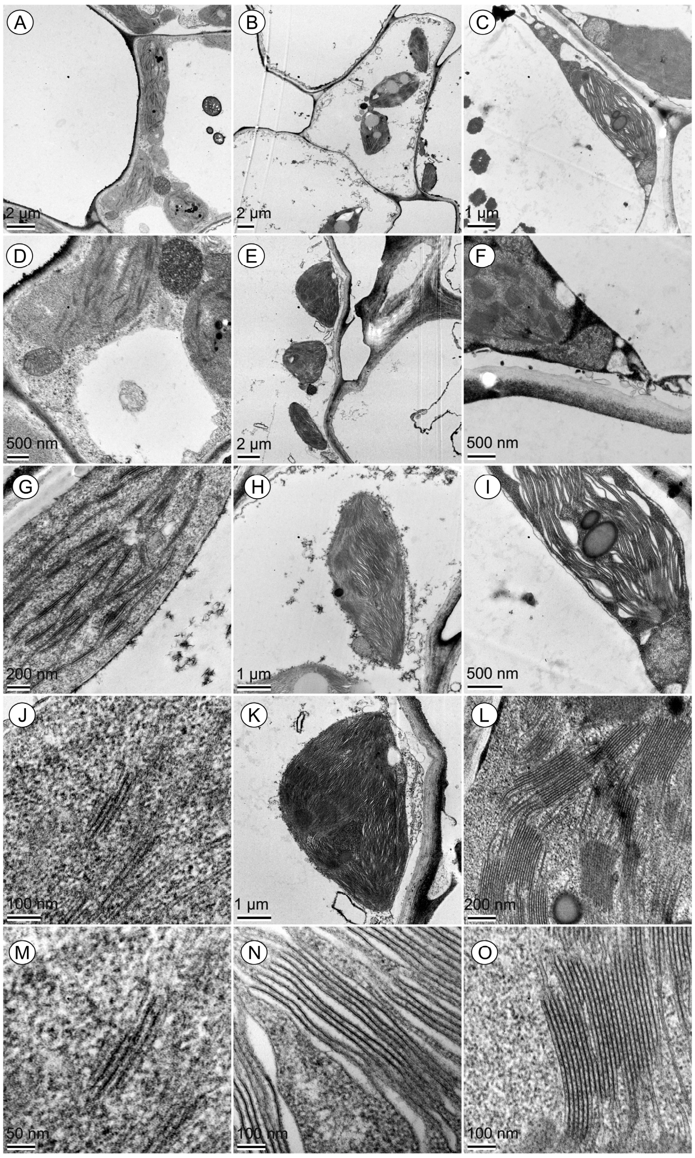

Figure S1. Transmission electron microscopy of chloroplasts in mesophyll cells of *Epipremnum aureum* leaves. Representative micrographs for (**A, D, G, J, M**) 'Neon', (**B, E, H, K, N**) 'Golden' and (**C, F, I, L, O**) 'Jade' phenotypes illustrate the general organisation of mesophyll cells and the spatial distribution of chloroplasts, together with progressively higher-magnification views of chloroplast morphology, thylakoid systems, grana stacking and membrane appression. Scale bars = 2 µm (**A,B,E**), 1 µm (**C,H,K**), 500 nm (**D,F,I**), 200 nm (**G,L**), 100 nm (**J,N,O**) and 50 nm (**M**), as indicated in each panel.
